# TNM stage in the Nordic Cancer Registries 2004–2016: Registration and availability

**DOI:** 10.2340/1651-226X.2024.35232

**Published:** 2024-05-07

**Authors:** Gerda Engholm, Frida E. Lundberg, Simon M. Kønig, Elínborg Ólafsdóttir, Tom B. Johannesen, David Pettersson, Nea Malila, Lina S. Mørch, Anna L. V. Johansson, Søren Friis

**Affiliations:** a Danish Cancer Institute, Danish Cancer Society, Copenhagen, Denmark; b Department of Medical Epidemiology and Biostatistics, Karolinska Institutet, Stockholm, Sweden; c Department of Oncology-Pathology, Karolinska Institutet, Stockholm, Sweden; d Danish Cancer Institute, Cancer Epidemiology and Surveillance, Danish Cancer Society, Copenhagen, Denmark; e Icelandic Cancer Registry, Reykjavik, Iceland; f Cancer Registry of Norway, Oslo, Norway; g Swedish Cancer Registry, National Board of Health and Welfare, Stockholm, Sweden; h Finnish Cancer Registry, Helsinki, Finland; i Danish Cancer Institute, Cancer and Medicine, Danish Cancer Society, Copenhagen, Denmark

**Keywords:** Cancer stage, TNM, Cancer registries, NORDCAN, relative survival

## Abstract

**Background and purpose:**

Stage at cancer diagnosis is an important predictor of cancer survival. TNM stage is constructed for anatomic solid cancer diagnoses from tumor size (T), nodal spread (N) and distant metastasis (M) and categorized in groups 0–I, II, II and IV. TNM stage is imperative in cancer diagnosis, management and control, and of high value in cancer surveillance, for example, monitoring of stage distributions. This study yields an overview of TNM availability and trends in stage distribution in the Nordic countries for future use in monitoring and epidemiologic studies.

**Material and methods:**

TNM information was acquired from the cancer registries in Denmark, Norway, Sweden, and Iceland during 2004–2016 for 26 cancer sites in the three former countries and four in Iceland. We studied availability, comparability, and distribution of TNM stage in three periods: 2004–2008, 2009–2013, and 2014–2016, applying a previously validated algorithm of ‘N0M0 for NXMX’. For cancers of colon, rectum, lung, breast, and kidney, we examined TNM stage-specific 1-year relative survival to evaluate the quality in registration of TNM between countries.

**Results:**

Denmark, Sweden, and Iceland exhibited available TNM stage proportions of 75–95% while proportions were lower in Norway. Proportions increased in Sweden over time but decreased in Denmark. One-year relative survival differed substantially more between TNM stages than between countries emphasizing that TNM stage is an important predictor for survival and that stage recording is performed similarly in the Nordic countries.

**Interpretation:**

Assessment and registration of TNM stage is an imperative tool in evaluations of trends in cancer survival between the Nordic countries.

## Background

Stage at cancer diagnosis is an important predictor of cancer survival [1, 2]. For solid tumors, stage of disease is mainly classified according to the Tumor-Nodal-Metastasis (TNM) classification system. The individual components, that is, T (tumor size), N (nodal spread), and M (metastasis), follow strict definitions, and the precise TNM stage is constructed from all three components and categorized in stages 0, I, II, II and IV [2]. Information on TNM stage is an imperative tool in cancer diagnosis and management [1, 2]. Moreover, the TNM stage is of high value in cancer control, surveillance, and research, for example, monitoring of trends in stage distribution at cancer diagnosis and monitoring and benchmarking of cancer incidence and survival [2, 3].

The nationwide cancer registries in the Nordic countries provide data for collaborative and comparative studies on cancer incidence and survival [4]. To facilitate access to Nordic cancer statistics, a publicly available database, NORDCAN, has been developed providing aggregate statistics on cancer incidence, cancer mortality, prevalence and survival in the Nordic countries [5–7]. The cancer registries in Denmark and Sweden initiated mandatory registration of TNM components in 2004, whereas TNM registration in Norway is voluntary and, in Iceland, TNM is only collected for a few cancer sites. In Finland, TNM information is reported, but not registered systematically.

Previous cancer surveillance studies have reported differences in cancer incidence and survival between the Nordic countries [8, 9]. To improve the understanding of the underlying causes for these differences, additional information on possible contributing factors, such as stage at cancer diagnosis, is warranted. Differences in survival could be because of differences in diagnostic delay, access to care (e.g. geographical disparities in access), stage registration, primary treatment, comorbidity and stage-specific survival. In consideration of these, quality data on TNM is highly valuable.

We examined how TNM information is acquired in the Nordic cancer registries and compared the availability, comparability, and distribution of TNM stage between the registries during 2004–2016. Changes in stage distribution over time, stage-shifts and time trends were evaluated. In addition for selected cancer sites (i.e. cancers of colon, rectum, lung, breast, and kidney), we estimated TNM stage-specific 1-year relative survival to evaluate the quality in registration of TNM between countries.

## Material and methods

### Acquisition of TNM data in the Nordic cancer registries

#### Denmark

Registration of TNM to the Danish Cancer Registry became mandatory in 2004 as part of modernization and automatization of the Cancer Registry [10]. Information in the automated Cancer Registry is derived from the Danish National Patient Registry [11] (primary source), Pathology Registry [12], Cause of Death Registry [13], and historical records in the Cancer Registry [10]. The recording of TNM is based on the highest reported values of the individual components (T, N, M) within 4 months from the cancer diagnosis. The basis of the determination of T, N, and M, that is, whether clinical or postsurgical/pathologic, was not registered during the study period of this article (and has become available only recently). If no registration of TNM is available in the Patient Registry, the Pathology Registry is used as a secondary source [12]. In Denmark, TNM information is also registered in cancer site-specific clinical cancer registries holding detailed information on cancer patients undergoing therapy, including diagnostic details (e.g. TNM), cancer management, and patient paths [14]. However, TNM data in the clinical registries are not available for recording in the Danish Cancer Registry.

#### Norway

In the Norwegian Cancer Registry, cancer stage is primarily registered as localized, regional, or metastatic based on electronic notifications to the Registry, performed mainly by clinicians. TNM, as clinical (cTNM), is voluntarily and thus only partly reported and registered. Since 2007, the Norwegian Cancer Registry has begun hosting an array of clinical cancer databases providing additional pathologic TNM (pTNM) registrations to the Registry [14].

#### Sweden

Information on cancer cases in Sweden is collected by six Regional Cancer Centres/Registries using the INCA reporting system, a registration platform used by all clinical cancer quality registries and six regional cancer registries in which new cancer cases are registered before they are sent to the Swedish Cancer Registry [15]. Since 2004, reporting of TNM to the National cancer registry has been mandatory for clinical departments and pathology units. Most of the TNM-registrations in the National Cancer Registry originate from one of about 30 clinical quality registries [14] and are subsequently transferred to the National Cancer Registry. However, for cancers not covered by a clinical quality register and for cancer cases that for other reasons are not reported to a quality registry, TNM is reported directly to one of the six Regional Registries. The clinical quality registries typically report both the pathologic and clinical TNM but only one value is transferred to the National Cancer Registry. The choice of which TNM record to transfer varies between cancer sites. Consequently, TNM-registrations in the National Cancer Registry represents a mixture of pathologic and clinical TNM varying between cancer sites, but with high completeness and validity [14, 15].

#### Iceland

TNM information in Iceland was collected manually by the Icelandic Cancer Registry for a few selected cancer sites, that is, colon, rectum, breast, and prostate. The information was based on pathological reports and hospital patient records. Since 2018, initiatives have been taken, in cooperation with Landspitali University Hospital, to establish a platform for TNM registration similar to the Swedish INCA system for several additional cancer sites [15–16].

#### Finland

The clinical notifications to the Finnish Cancer Registry include a systematic feature for reporting cancer stage as localized, regional, or metastatic, supplemented by a ‘free text format’ option to report TNM. Thus, the registration of stage has been based only on the former and the TNM information just kept in the free text format. cTNM has been the preferred way to report stage since 2015, however, the information is missing in most notifications. The main source of notifications to the cancer registry derives from pathology reports, however, no systematic way of reporting and registration in The Finnish Cancer Registry have been established. To obtain TNM in a valid format from the ‘free text’, all text fields would have to be checked and re-coded. This would have to be predominantly manually, as the free text may contain empty spaces, Roman or Arabic numbers, letters, missing data, etc.

## Study data

This study included individual-level cancer data delivered from the Cancer Registries to update the NORDCAN database with 2016 data [3]. Thus, data on incident cancers including TNM information were retrieved from the national cancer registries in Denmark, Norway, Sweden, and Iceland. No TNM data were available from Finland. We selected cancer cases from 26 cancer entities diagnosed in 2004–2016 (Supplementary Table 1). Gynecological cancers were not included in Norway and Sweden because in these countries stage was reported only according to the Federation of Gynecology and Obstetrics (FIGO) classification and was not available in the datasets. Cancer entities were defined according to the present version of NORDCAN [6].

In all four study countries (i.e. Denmark, Norway, Sweden, and Iceland), TNM registrations were coded by clinicians according to Union for International Cancer Control (UICC) TNM manuals version 5, 6, or 7 [17–19]. The TNM manuals specify coding of TNM components and conversions to TNM stage according to specific anatomic diagnostic groups (TNM sites) using the International Classification of Diseases for Oncology version 3 (ICD-O-3) topography groups. We specified the TNM sites according to ICD-10 (Supplementary Table 2). The conversion was performed at the former NORDCAN secretariat in Denmark according to a Statistical Analysis System (SAS) macro developed by the first author (GE). The UICC version 7 conversion to TNM stage was used to facilitate optimal comparison over time [19]. Some TNM sites (i.e. cancers of thyroid, thymus, bone and soft tissue, eye, and non-melanoma skin cancer) were not included as their conversion required information (e.g. grade) not available in the cancer registries.

In Denmark and Sweden, no information was available on whether the TNM registrations were based on cTNM or pTNM criteria, whereas this was specified in Iceland and Norway for some cases. In our conversion, we preferred pathologic TN (pTN) over clinical TN (cTN) while the highest value of clinical M (cM) and pathologic M (pM) was used. For breast cancer in Norway, information on TNM stage was supplemented from the clinical cancer database.

In tabulations of stage distributions, we used the cancer entity groups from NORDCAN [6] with underlying information on stage from TNM sites.

According to the TNM manuals [17–19], all three TNM components (i.e. T, N and M) are necessary to determine the TNM stage (except for TXNXM1). In the main analyses of the present study, however, we assumed that missing information on N or M (NX and MX) could be interpreted as N0 and M0, as documented in our previous study [3]. Herein, we showed that application of the algorithm was reasonable and yielded no major changes in 1-year stage-specific survival estimates, while substantially increasing the proportion of cases with available TNM.

We categorized the TNM stage as 0, I, II, III, and IV. For visibility and convenience, we combined TNM stages 0 and I (0-I). Moreover, we defined two categories of missing information: ‘No info’ including cases with no information at all or reporting of ‘unknown TNM’ (TXNXMX), and ‘Partly info’ including cases with valid T and/or N value but unknown M (MX), and cases with M0 but a non-valid T and/or N value.

## Statistical methods

For the selected cancer entities in the four study countries during 2004–2016, we calculated the number of cases, proportions with available TNM stage and stage distribution using both the official definition of TNM stage (version 7) and applying the assumption of ‘N0M0 for NXMX’ [3]. We estimated results for three calendar periods of diagnosis: 2004–2008, 2009–2013, and 2014–2016.

For each calendar period, we estimated 1-year age-standardized relative survival for colon, rectum, lung, breast, and kidney cancer, with follow-up for death through 2017, using the Pohar Perme method approach with relative individual age weights [20] according to an adapted version of the International Cancer Survival Standard 1 (ICSS1) [9]. Analyses were performed ignoring sex and TNM stage and also separately for stage-specific groups and the missing TNM group. We used the strs stpp command [21] in Stata (StataCorp. 2021. Stata Statistical Software: Release 17. College Station, TX: StataCorp LLC) to estimate relative survival and 95% confidence intervals (CI), with the indweight option for individual weights [21].

We preferred 1-year relative survival to longer periods of survival because the former better reflects consequences of stage at diagnosis and is less influenced by differences between countries in cancer therapy. Besides, this also enabled survival calculations for the latest period 2014–2016.

In the survival estimations, we excluded cases diagnosed by death certificate alone or as incidental finding at autopsy, whereas these cases were included in the ‘No info’ category in tabulations of stage distributions.

## Results

We identified and tabulated 1,128,852 incident cases according to 26 selected NORDCAN cancer entities during 2004–2016 in Denmark, Norway, and Sweden, and Iceland (4 entities) (Supplementary Table 1).

The TNM stage for each cancer case was determined according to 31 TNM sites using the description in the TNM manuals. The information on numbers and ICD-10 codes are shown in Supplementary Table 2.

Table 1 presents proportions of cases with available TNM stage by country, cancer entity, and period applying the algorithm for missing information (‘N0M0 for NXMX’). We observed high (75–95%) proportions with available TNM stage for most cancer entities in Denmark, Sweden, and Iceland, whereas Norway generally exhibited lower proportions. Proportions of available TNM stage decreased in Denmark during the study period and by more than 10% percentage points from 2009–2013 to 2014–2016 for several cancer entities. In contrast, proportions increased in Sweden during the entire study period for most cancer entities. No major variation over time was seen in Norway.

**Table 1:** Proportions of cancer cases with available TNM-stage using the ‘N0M0 for NXMX’ assumption for 26 entities over three time periods, 2004–2008, 2009–2013 and 2014–2016, in Denmark, Norway, Sweden, and Iceland.

Entity	Denmark			Norway			Sweden			Iceland		
	2004–08	2009–13	2014–16	2004–08	2009–13	2014–16	2004–08	2009–13	2014–16	2004–08	2009–13	2014–16
Lip	91	94	91	47	38	30	85	95	92	.	.	.
Oral cavity	89	79	66	71	65	66	89	98	98	.	.	.
Salivary glands	86	78	62	65	59	58	83	96	97	.	.	.
Oropharynx	89	84	67	78	80	78	89	99	99	.	.	.
Nasopharynx	87	77	64	75	63	76	82	94	97	.	.	.
Hypopharynx	90	82	73	71	73	78	86	98	99	.	.	.
Esophagus	81	84	73	37	45	41	68	84	88	.	.	.
Stomach	84	84	77	38	47	44	68	80	84	.	.	.
Small intestine	82	77	76	36	41	47	52	64	67	.	.	.
Colon	89	85	81	61	81	85	87	95	93	93	92	89
Rectum	88	85	79	64	67	75	82	92	90	.	70	88
Anus	73	76	41	51	50	31	40	40	52	.	.	.
Liver	66	69	63	22	28	34	45	71	81	.	.	.
Gallbladder	68	75	68	34	40	47	65	74	77	.	.	.
Pancreas	80	80	73	33	41	37	64	81	84	.	.	.
Nose, sinuses	84	79	65	67	50	47	78	91	89	.	.	.
Larynx	94	90	66	74	63	63	90	98	97	.	.	.
Lung	94	97	96	63	72	78	92	96	96	.	.	.
Pleura	87	90	80	35	42	38	50	52	56	.	.	.
Breast	97	95	89	96	95	98	83	96	96	97	97	95
Prostate	85	86	80	89	88	83	94	96	96	95	96	93
Testis	95	89	80	53	45	34	85	86	94	.	.	.
Penis etc.	82	75	70	50	55	28	85	88	81	.	.	.
Kidney	88	84	89	63	65	49	87	95	96	.	.	.
Bladder etc.	93	94	95	65	45	45	82	88	93	.	.	.
Melanoma, skin	92	94	92	25	17	95	79	96	97	.	.	.

Study of TNM stage at diagnosis in the Nordic countries 2004–2016.

Comparing the ‘N0M0 for NXMX’ assumption and the official definition of TNM stage, TNM stage availability increased substantially (>40%) for prostate and bladder cancer (Supplementary Tables 23 and 26). Generally, TNM stage availability increased by 10–20 percentage points. Slightly lower increases were observed for Norway (Supplementary Tables 3–28).

**Figure 1:**
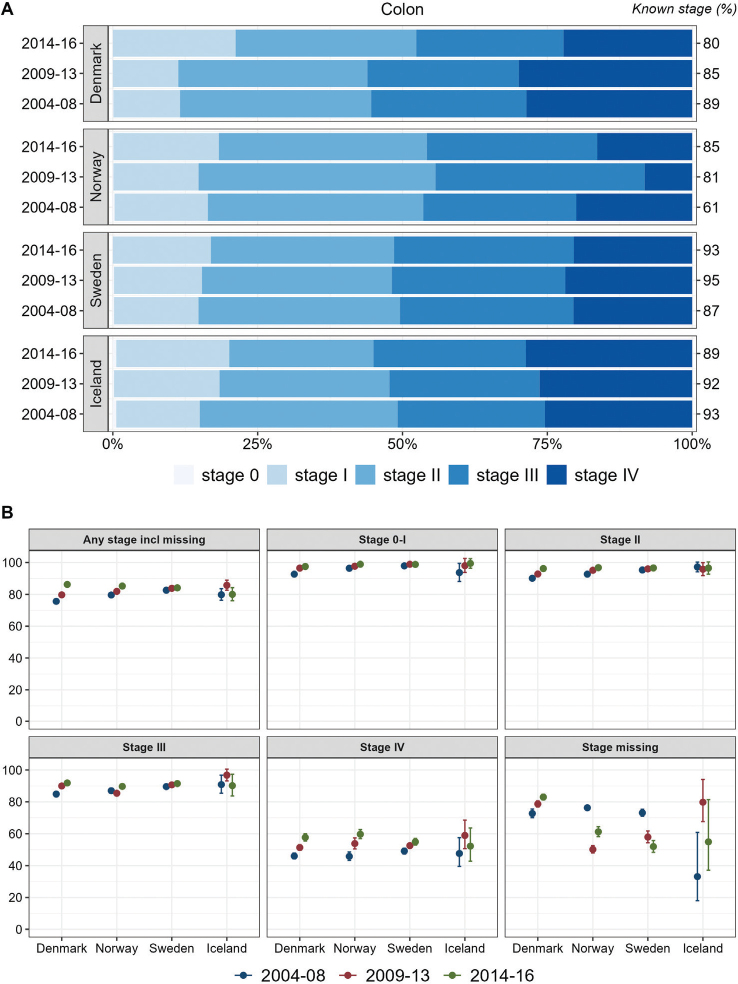
Colon cancer. Time trends for TNM stages in Denmark, Norway, Sweden, and Iceland during 2004–2008, 2009–2013, and 2014–2016. (A) Stage distribution among cases with available TNM stage. (B) 1-year age-standardized relative survival, according to groups of all cases, stages 0-I, II, III, IV, and stage missing.

For colon cancer, the stage distribution was stable in Sweden in the study period. In Iceland, the proportions of stage I and IV increased over the years. In Denmark, higher proportions were observed for advanced stages (III–IV), but in the most recent period, 2014–2016, the proportion of stage I was higher, and the proportion of stage IV had decreased and was comparable to that in Sweden. In Norway, the proportion with stage IV colon cancer was lower than in the other countries, especially in 2009–2013. Stage-specific 1-year relative survival of colon cancer increased over time in all countries, except for stage III in Norway, and survival estimates were comparable between countries within stages (Figure 1). For the group of cases with missing stage records, survival was highest in Denmark and increased over time while it decreased in Norway and Sweden.

**Figure 2:**
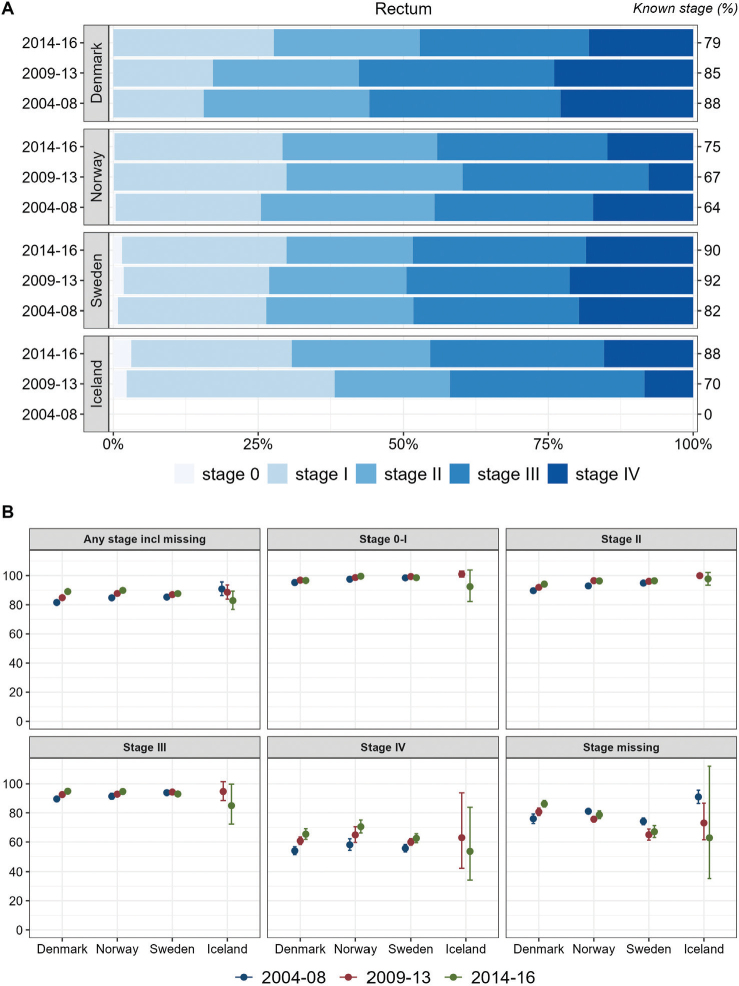
Rectum cancer. Time trends for TNM stages in Denmark, Norway, Sweden, and Iceland during 2004–2008, 2009–2013, and 2014–2016. (A) Stage distribution among cases with available TNM stage. (B) 1-year age-standardized relative survival according to groups of all cases, stages 0-I, II, III, IV, and stage missing.

Results for rectal cancer (Figure 2) were similar to those for colon cancer, except for a tendency toward decreasing survival over time in Iceland. In the other three study countries, stage-specific 1-year survival increased over time.

**Figure 3:**
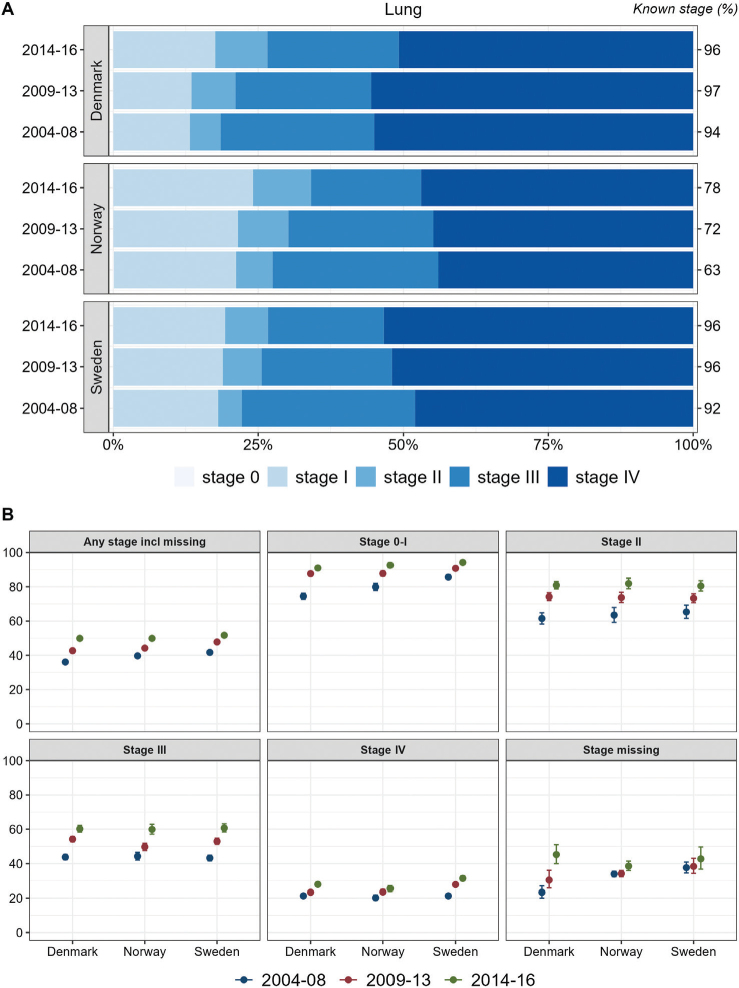
Lung cancer. Time trends for TNM stages in Denmark, Norway, and Sweden during 2004–2008, 2009–2013, and 2014–2016. (A) Stage distribution among cases with available TNM stage. (B) 1-year age-standardized relative survival according to groups of all cases, stages 0–I, II, III, IV, and stage missing.

Overall, about half of the lung cancer cases were recorded with stage IV at diagnosis, although with a slightly lower proportion in Norway. In all countries, the proportions of stage I and II increased slightly during the study period whereas stage III decreased. One-year relative survival increased over time for each stage and for all stages together, with highest stage I and stage IV survival observed in Sweden (Figure 3).

**Figure 4:**
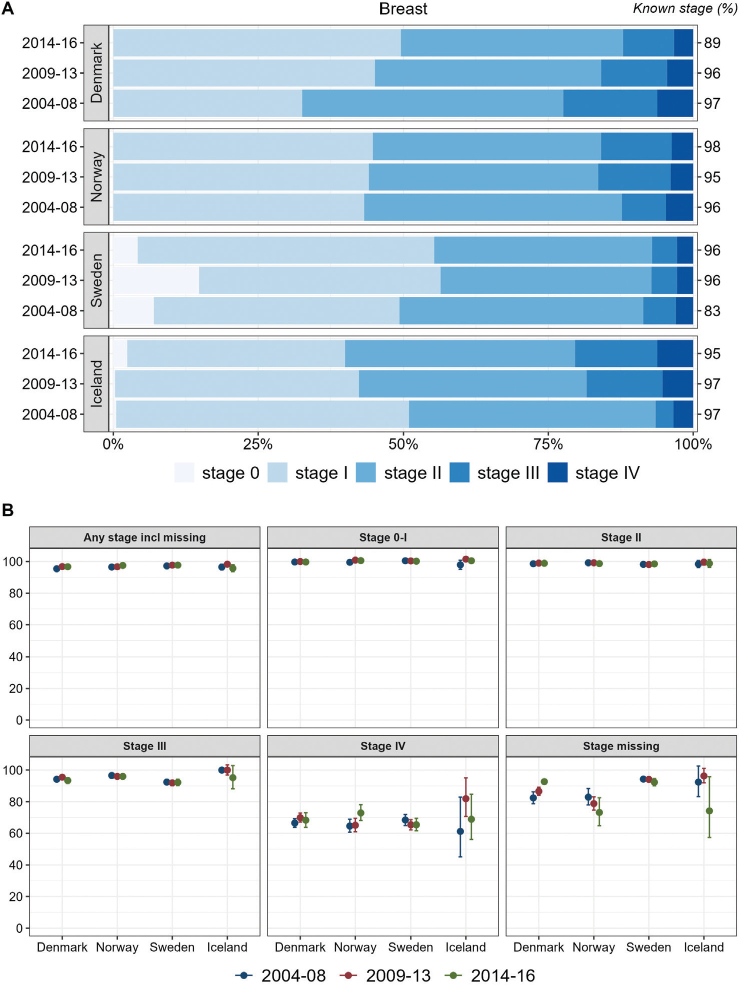
Breast cancer. Time trends for TNM stages in Denmark, Norway, Sweden, and Iceland during 2004–2008, 2009–2013, and 2014–2016. (A) Stage distribution among cases with available TNM stage. (B) 1-year age-standardized relative survival according to groups of all cases, stages 0–I, II, III, IV, and stage missing.

For breast cancer, stage IV proportions were around 5% in all countries and periods. Denmark exhibited a more unfavorable distribution in 2004–2008 than the other Nordic countries. Sweden had a more favorable stage distribution in all years and especially a high proportion of stage 0 (Figure 4A). The proportions of stage 0–I, however, increased in both Sweden and Denmark during 2009–2013. In Iceland, the stage distribution became less favorable during the study period, with higher proportions of advanced disease (III/IV). Sweden had the lowest proportions of cancer cases with stage II and III, but lower stage II and III survival than the other countries across all periods (Figure 4).

**Figure 5:**
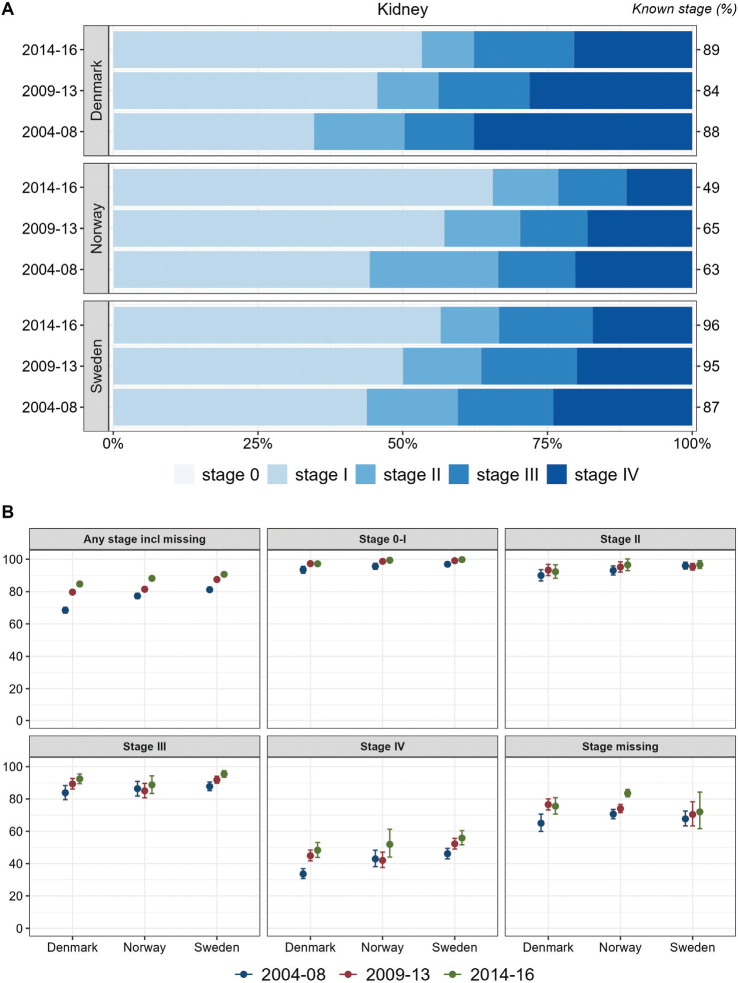
Kidney Cancer. Time trends for TNM stages in Denmark, Norway, and Sweden during 2004–2008, 2009–2013, and 2014–2016. (A) Stage distribution among cases with available TNM stage. (B) 1-year age-standardized relative survival according to groups of all cases, stages 0–I, II, III, IV, and stage missing.

Stage distributions for kidney cancer improved with higher proportions of low stage over time. Initially in the study period, Denmark exhibited the lowest proportion of low stage kidney cancer. In the most recent period, Norway exhibited the most favorable stage distribution followed by Sweden and Denmark. In all countries, stage-specific survival improved over time, with the highest survival observed in Sweden, except for cancer cases with missing stage for whom survival was lowest in Sweden (Figure 5).

For all five cancer sites, cases with no available TNM stage exhibited larger differences in survival between the four Nordic study countries.

## Discussion

In general, we found that differences in survival between TNM stages were much larger than differences in stage-specific survival between countries. Thus, our study emphasizes that TNM stage is an important predictor for survival and that stage recording is used in a comparable way between the Nordic countries. The large differences in survival between groups with missing TNM stage reflect the variation in completeness of TNM registration between countries. The higher proportion of patients with available TNM stage in Denmark and Sweden is likely due to the mandatory registration, while the lower completeness in Norway is consistent with the voluntary registration of TNM and later establishment of clinical cancer databases. In Sweden, the proportion of available TNM stage increased over time, while the opposite and unfavorable trend was seen in Denmark.

The improvements in stage-specific survival, notably for lung cancer and stage IV colorectal and kidney cancer, are likely due to improved therapy. Therapeutic improvements have been suggested by national clinical/quality cancer groups with responsibility of preparing clinical guidelines according to the most recent evidence [14]. Such initiatives have been taken in all Nordic countries and differences in survival are therefore not likely to be due to differences in the standard cancer therapy. Uniformity in cancer management in the Nordic countries is also supported by the similar 1-year stage-specific survival between the countries for the study cancer sites (i.e. colon, rectum, lung, breast, and kidney). Differences in stage distribution are, however, likely to induce differences in overall cancer survival. Assessment and registration of TNM stage is thus an imperative tool in evaluations of trends in cancer survival between the Nordic countries.

Earlier diagnosis and introduction of screening lead to a more favorable stage distribution (stage shift). National screening for breast cancer was introduced in Denmark during 2007–2009 as the last Nordic country, but Denmark was the first Nordic country to initialize general colorectal cancer screening in 2014. Screening initiatives are typically followed by an increase in incidence followed by return to the previous or a lower level but with an improved stage distribution with higher proportions of low-stage cancer. This pattern was documented and discussed for breast, colon, and rectum cancer in our previous study [9]. Another consequence of improved diagnostic intensity is more frequent incidental finding of kidney or other cancers as part of diagnostic work-up and imaging for non-cancer diseases. This reflects an international increasing trend for kidney cancer [22] and other cancers, for example, in Denmark in 2015–2016, the proportion of kidney cancer cases diagnosed as accidental findings was 46% [23].

A recent Nordic study reported that cancer survival has improved in Denmark up to 2016 reaching a level similar to that of the other Nordic countries [9]. Establishment of a revised (second) national cancer plan in Denmark in 2004, and a political decision in 2007 to consider cancer as an acute disease, was followed by accelerated and accentuated cancer patient pathways reducing waiting time to diagnosis and treatment, ensuring standardized clinical work-up programs, and consequently improving survival [24, 25]. These initiatives are considered to have substantially contributed to the improved stage distribution in Denmark for most cancer types as well as the improvements in survival [6, 9]. Accelerated cancer patient pathways were also established and implemented in 2015 in Norway and Sweden. Norwegian studies evaluating the implementation of cancer patient pathways for colorectal, lung, breast and prostate cancer patients showed decreased waiting time to treatment and improved stage distribution from 2007 to 2016 [26].

We observed an improved TNM availability during the study period in Sweden, but a decrease in Denmark. For colorectal cancer in Denmark, part of the higher proportion with missing TNM stage and higher survival for the group with missing TNM stage, especially in the last period, was due to about 5% of cases having M0 but missing T and/or N and thus with missing TNM stage (not shown in tables). From 2019, Danish clinicians were allowed to postpone TNM registration to the hospital registry/cancer registry up to 1 month after diagnosing an incident cancer to secure registration of additional detail from the clinical work-up. Such prolongation in the process of reporting with subsequent harvesting of TNM registrations from the Patient Register, might have contributed to the decrease in available TNM registration in Denmark. Part of the missing information has been supplemented by information from the Pathology Register, but a more efficient reminder procedure is planned.

In Sweden, a considerable proportion of breast cancers were reported as TNM stage 0 (>16% in 2009–2013, Figure 4A), and Sweden had higher proportions in stage 0–I and II than Denmark and Norway. Screening often leads to earlier diagnosis with lower stage and some overdiagnosis. The age interval for screening in Sweden is broader (40–74 years) than in Denmark and Norway (50–69 years). Besides, the Swedish stage information was mainly based on clinical information where non-palpable tumors were coded as T0 [27]. This might induce the ‘Will Rogers phenomenon’ with some cases being placed in lower stage than would be the case in other countries [28]. We found lower stage-specific survival for stage III in Sweden than in Denmark and Norway and slightly lower, non-significant survival in stage II.

We applied the TNM version 7 conversions to TNM stage for the entire study period, 2004–2016 [19]. The year of change from TNM versions 6 to 7 in clinical practice varied slightly between countries, yet this has likely not resulted in substantial differences between countries. For lung cancer, though, the definitions of T and N changed between versions 6 and 7 and might have influenced the general pattern of TNM stage distribution for lung cancer over time [18, 19].

The ‘N0M0 for NXMX’ assumption generally increased the number of patients with available TNM stage and made TNM distribution more comparable between countries [3]. In the Supplementary Tables, TNM distributions according to the official TNM stage conversion and to the algorithm of ‘N0M0 for NXMX’ are presented for the 26 study cancer entities and periods (i.e. 2004–2008, 2009–2013, and 2014–2016). Although the ‘N0M0 for NXMX’ assumption may be reasonable, more complete registration of TNM would be preferable, and we urge clinicians to report complete TNM information to the cancer registries in order to improve the quality of cancer surveillance in the Nordic countries. In this context, it should be acknowledged that many patients do not undergo investigations beyond the T or T+N stage, that is, a comprehensive evaluation to determine the complete TNM stage is often not performed. In such cases, a general rule according to the TNM manual is that if in doubt of specific categories, the lower option should be chosen.

Limitations of our study are that we presented results for a limited number of cancer sites and that we did not provide results, for example, stage differences, in subgroups of the patient populations (e.g. sex and age). Moreover, our study was limited to data between 2004 and 2016, and as such did not fully evaluate the impact of the most recent developments in cancer diagnostics and therapy, for example, MR in diagnosis of prostate cancer and immunotherapy against an increasing number of target cancer sites.

In conclusion, our results emphasize that TNM stage is an important tool for cancer surveillance and management, and that high-quality harmonized registration and reporting of TNM are essential. For cancer surveillance and international benchmarking, monitoring of stage distribution over time is an efficient and up-to-date tool when evaluating implementation of initiatives for early diagnosis.

## Supplementary Material

TNM stage in the Nordic Cancer Registries 2004–2016: Registration and availability

## Data Availability

Individual person information is not freely available due to GDPR legislation. Tabulated data for the cases included (by sex, age, cancer site, year of diagnoses, country but not TNM) can be seen in the publicly available Nordic cancer statistics database NORDCAN, https://nordcan.iarc.fr/en. Application for access to individual data from each of the countries can be made to the National Cancer Registries in Denmark, Norway, Sweden, and Iceland.
