# Supplementary material for: TNM stage in the Nordic Cancer Registries 2004–2016: Registration and availability
Source: Acta Oncol. 2024 May 7;63:35232. doi: 10.2340/1651-226X.2024.35232 (PMC11332511; doi:10.2340/1651-226X.2024.35232)
Supplement: TNM stage in the Nordic Cancer Registries 2004–2016: Registration and availability [file AO-63-35232-s1.pdf]

Supplementary material has been published as submitted. It has not been copyedited or typeset by Acta Oncologica.

## Supplementary tables for

# TNM stage in the Nordic Cancer Registries 2004-2016: Registration and availability

Page 2 – Table 1. Study of TNM stage in the Nordic countries 2004-2016. NORDCAN entities with ICD10 codes and included number of cases by country.

Page 3 – Table 2. Study of TNM stage in the Nordic countries 2004-2016. TNM-sites with ICD10 codes and included number of cases by country.

Pages 4-16 – Tables 3-28, Time trends for TNM stages in Denmark, Norway, Sweden, and Iceland over 2004-2008, 2009-2013 and 2014-2016

Page 4 – Table 3. Lip cancer, Table 4. Oral cavity cancer

Page 5 – Table 5. Salivary glands cancer, Table 6. Oropharynx cancer

Page 6 – Table 7. Nasopharynx cancer, Table 8. Hypopharynx cancer

Page 7 – Table 9. Oesophagus cancer, Table 10. Stomach cancer

Page 8 – Table 11. Small intestine cancer, Table 12. Colon cancer

Page 9 – Table 13. Rectum cancer, Table 14. Anal cancer

Page 10 – Table 15. Liver cancer, Table 16. Gallbladder cancer

Page 11 – Table 17. Pancreas cancer, Table 18. Nose. Sinus cancer

Page 12 – Table 19. Larynx cancer, Table 20. Lung cancer

Page 13 – Table 21. Pleura cancer, Table 22. Breast cancer

Page 14 – Table 23. Prostate cancer, Table 24. Testis cancer

Page 15 – Table 25. Penis etc. cancer, Table 26. Kidney cancer

Page 16 – Table27. Bladder cancer, Table 28. Melanoma skin cancer

Supplementary Table 1. Study of TNM stage in the Nordic countries 2004-2016. NORDCAN entities with ICD10 codes and included number of cases by country.

| | | Number of cases by country | | | |
|---|---|---|---|---|---|
| NORDCAN entity | ICD10 | Denmark | Norway | Sweden | Iceland |
| Lip | C00.0-C00.2, C00.6, C00.8-C00.9 | 432 | 1154 | 1868 | |
| Oral Cavity | C00.3-C00.5, C02-C04 C05.0, C05.8-C05.9, C06 | 4317 | 2294 | 4728 | |
| Salivary glands | C07-C08 | 767 | 655 | 1363 | |
| Oropharynx | C01, C05.1-C05.2, C09, C10.0, C10.2-C10.9, C14.0, C14.2-C14.8 | 4250 | 1983 | 3884 | |
| Nasopharynx | C11 | 312 | 181 | 412 | |
| Hypopharynx | C12-C13 | 1249 | 333 | 765 | |
| Oesophagus | C15 | 6048 | 3043 | 5734 | |
| Stomach | C16 | 7266 | 6461 | 11295 | |
| Small intestine | C17 | 1518 | 1795 | 3470 | |
| Colon | C18 | 38144 | 33512 | 50555 | 1394 |
| Rectum | C19-C20 | 19805 | 16229 | 25924 | 517 |
| Anus | C21 | 1561 | 935 | 1959 | |
| Liver | C22 | 4612 | 2550 | 7529 | |
| Gallbladder | C23-C24 | 2575 | 2048 | 5075 | |
| Pancreas | C25 | 12108 | 9348 | 14269 | |
| Nose, sinuses | C30-C31 | 901 | 575 | 910 | |
| Larynx | C10.1, C32 | 3325 | 1518 | 2315 | |
| Lung | C33-C34 | 57209 | 35705 | 48975 | |
| Pleura | C38.4, C45.0, C45.9 | 1481 | 1008 | 1560 | |
| Breast | C50 | 60255 | 38865 | 88992 | 2618 |
| Prostate | C61 | 55082 | 58882 | 128652 | 2822 |
| Testis | C62 | 3878 | 3773 | 4242 | |
| Penis etc. | C60, C63 | 805 | 644 | 1257 | |
| Kidney | C64 | 10072 | 9395 | 13778 | |
| Bladder etc | C65-C68, D09.0-D09.1, D30.1-D30.9, D41.1-D41.9 | 25773 | 18561 | 34526 | |
| Melanoma, skin | C43 | 25444 | 20343 | 36485 | |
| Total | | 349189 | 271790 | 500522 | 7351 |

Supplementary Table 2. Study of TNM stage in the Nordic countries 2004-2016. TNM-sites with ICD10 codes and included number of cases by country.

| | | Number of cases by country | | | |
|---|---|---|---|---|---|
| TNM-sites | ICD10 | Denmark | Norway | Sweden | Iceland |
| Lip | C00.0-2,5-9 | 432 | 1154 | 1868 | |
| Oral cavity | C00.3-4,C02-04,C05.0,8-9, C06 | 4317 | 2294 | 4728 | |
| Oropharynx, p16-neg | C01,C05.1-2, C09,C10.0,2-9 | 4230 | 1950 | 3822 | |
| Nasopharynx | C11 | 312 | 181 | 412 | |
| Hypopharynx | C12-14 | 1269 | 366 | 827 | |
| Nasal Cavities and sinuses | C30-31 | 901 | 575 | 910 | |
| Larynx | C32+C10.1 | 3325 | 1518 | 2315 | |
| Salivary glands | C07-08 | 767 | 655 | 1363 | |
| Oesophagus | C15-16.0 | 9424 | 4649 | 8700 | |
| Stomach | C16.1-9 | 3890 | 4855 | 8329 | |
| Small intestine | C17 | 1518 | 1795 | 3470 | |
| Appendix | C18.1 | 616 | 843 | 770 | 26 |
| Colon and rectum | C18,0,2-9, C19-20 | 57333 | 48898 | 75709 | 1885 |
| Anal canal | C21 | 1561 | 935 | 1959 | |
| Liver, hepatocellular carc. | C22.0,7,9 | 3683 | 1855 | 5873 | |
| Liver, intrahepatic bile ducts | C22.1 | 849 | 654 | 1618 | |
| Gallbladder | C23 | 546 | 489 | 2232 | |
| Distal extrahepatic bile ducts | C24.0,8-9 | 1474 | 1106 | 2200 | |
| Ampulla of vater | C24.1 | 555 | 453 | 643 | |
| Pancreas | C25 | 12108 | 9348 | 14269 | |
| Lung | C34 | 57171 | 35665 | 48920 | |
| Pleura | C38.4+C45.0,9 | 1481 | 1008 | 1560 | |
| Malignant melanoma, skin | C43 | 25444 | 20343 | 36485 | |
| Breast | C50 | 60255 | 38865 | 88992 | 2618 |
| Penis | C60 | 745 | 566 | 1201 | |
| Prostate | C61 | 55082 | 58882 | 128652 | 2822 |
| Testis | C62 | 3878 | 3773 | 4242 | |
| Kidney | C64 | 10072 | 9395 | 13778 | |
| Renal pelvis and ureter | C65-66 | 1256 | 1480 | 2362 | |
| Urinary bladder | C65-67,D09.0-1,D30.1-9 | 24422 | 16904 | 31744 | |
| Urethra | C68 | 95 | 177 | 420 | |
| Not in a TNM-site | | 178 | 159 | 149 | 0 |
| Total | | 349189 | 271790 | 500522 | 7351 |

Supplementary Table 3. Lip cancer. Time trends for TNM stages in Denmark, Norway, and Sweden over 2004-2008, 2009-2013 and 2014-2016

| **Lip** | Official TNM | | | Using N0M0 for NXMX | | |
|---|---|---|---|---|---|---|
| | Denmark | Norway | Sweden | Denmark | Norway | Sweden |
| **2004-09** | | | | | | |
| No info | 4.9 | 50.9 | 13.4 | 4.9 | 50.9 | 13.4 |
| Partly | 10.3 | 6.9 | 12.8 | 3.6 | 2.1 | 2.0 |
| TNM-inf | 84.8 | 42.2 | 73.7 | 91.5 | 46.9 | 84.6 |
| 0-I | 84.1 | 78.0 | 87.3 | 84.3 | 78.5 | 87.1 |
| II | 12.7 | 13.8 | 9.4 | 12.3 | 13.0 | 9.6 |
| III | 1.1 | 1.3 | 2.0 | 1.5 | 2.3 | 2.0 |
| IV | 2.1 | 6.9 | 1.2 | 2.0 | 6.2 | 1.2 |
| **2009-13** | | | | | | |
| No info | 3.9 | 61.0 | 5.2 | 3.9 | 61.0 | 5.2 |
| Partly | 15.8 | 4.5 | 5.3 | 2.0 | 1.4 | 0.1 |
| TNM-inf | 80.3 | 34.6 | 89.6 | 94.1 | 37.6 | 94.7 |
| 0-I | 77.0 | 82.4 | 82.4 | 74.8 | 81.1 | 82.6 |
| II | 14.8 | 11.2 | 12.6 | 17.5 | 10.8 | 12.5 |
| III | 3.3 | 5.3 | 2.6 | 3.5 | 6.5 | 2.6 |
| IV | 4.9 | 1.2 | 2.4 | 4.2 | 1.6 | 2.3 |
| **2014-16** | | | | | | |
| No info | 3.5 | 69.8 | 7.9 | 3.5 | 69.8 | 7.9 |
| Partly | 22.8 | 3.5 | 6.3 | 5.3 | 0 | 0.2 |
| TNM-inf | 73.7 | 26.7 | 85.8 | 91.2 | 30.2 | 91.9 |
| 0-I | 90.5 | 68.4 | 79.3 | 90.4 | 69.8 | 80.0 |
| II | 7.1 | 22.4 | 16.1 | 7.7 | 22.1 | 15.7 |
| III | 2.4 | 6.6 | 3.0 | 1.9 | 5.8 | 2.8 |
| IV | 0 | 2.6 | 1.6 | 0 | 2.3 | 1.5 |

Supplementary Table 4. Oral cavity cancer. Time trends for TNM stages in Denmark, Norway, and Sweden over 2004-2008, 2009-2013 and 2014-2016

| **Oral cavity** | Official TNM | | | Using N0M0 for NXMX | | |
|---|---|---|---|---|---|---|
| | Denmark | Norway | Sweden | Denmark | Norway | Sweden |
| **2004-09** | | | | | | |
| No info | 9.4 | 27.3 | 10.1 | 9.4 | 27.3 | 10.1 |
| Partly | 18.9 | 8.2 | 12.2 | 1.6 | 1.7 | 1.0 |
| TNM-inf | 71.8 | 64.5 | 77.8 | 89.1 | 71.0 | 89.0 |
| 0-I | 24.5 | 26.9 | 27.8 | 24.7 | 26.1 | 27.0 |
| II | 20.0 | 24.2 | 23.6 | 19.8 | 23.6 | 24.7 |
| III | 19.0 | 10.4 | 14.5 | 18.9 | 10.7 | 14.1 |
| IV | 36.5 | 38.5 | 34.1 | 36.6 | 39.7 | 34.2 |
| **2009-13** | | | | | | |
| No info | 18.9 | 34.2 | 2.2 | 18.9 | 34.2 | 2.2 |
| Partly | 14.8 | 5.5 | 1.7 | 2.0 | 0.7 | 0.1 |
| TNM-inf | 66.4 | 60.3 | 96.2 | 79.1 | 65.1 | 97.7 |
| 0-I | 26.1 | 32.6 | 33.7 | 26.5 | 30.9 | 33.8 |
| II | 17.7 | 20.2 | 22.4 | 18.5 | 20.8 | 22.4 |
| III | 18.8 | 9.6 | 10.2 | 18.5 | 10.0 | 10.3 |
| IV | 37.4 | 37.5 | 33.6 | 36.4 | 38.3 | 33.6 |
| **2014-16** | | | | | | |
| No info | 32.0 | 32.3 | 2.1 | 32.0 | 32.3 | 2.1 |
| Partly | 11.5 | 4.4 | 0.5 | 2.5 | 1.3 | 0.2 |
| TNM-inf | 56.6 | 63.3 | 97.3 | 65.5 | 66.5 | 97.6 |
| 0-I | 31.2 | 27.5 | 30.2 | 32.9 | 26.9 | 30.3 |
| II | 19.4 | 23.3 | 22.4 | 18.9 | 23.1 | 22.4 |
| III | 13.5 | 10.1 | 10.2 | 13.6 | 10.1 | 10.2 |
| IV | 35.9 | 39.1 | 37.2 | 34.6 | 39.9 | 37.1 |

Supplementary Table 5. Salivary glands cancer. Time trends for TNM stages in Denmark, Norway, and Sweden over 2004-2008, 2009-2013 and 2014-2016

| **Salivary glands** | Official TNM | | | Using N0M0 for NXMX | | |
|---|---|---|---|---|---|---|
| | Denmark | Norway | Sweden | Denmark | Norway | Sweden |
| **2004-09** | | | | | | |
| No info | 8.3 | 34.5 | 15.4 | 8.3 | 34.5 | 15.4 |
| Partly | 19.2 | 8.0 | 10.3 | 5.3 | 1.0 | 2.1 |
| TNM-inf | 72.5 | 57.5 | 74.3 | 86.4 | 64.5 | 82.5 |
| 0-I | 31.2 | 24.3 | 31.3 | 30.1 | 24.0 | 30.7 |
| II | 19.8 | 31.3 | 23.5 | 23.1 | 30.2 | 23.4 |
| III | 16.1 | 14.8 | 17.7 | 16.6 | 14.7 | 18.2 |
| IV | 32.8 | 29.6 | 27.4 | 30.1 | 31.0 | 27.7 |
| **2009-13** | | | | | | |
| No info | 18.2 | 37.2 | 2.9 | 18.2 | 37.2 | 2.9 |
| Partly | 13.0 | 9.6 | 2.5 | 3.9 | 4.2 | 0.7 |
| TNM-inf | 68.8 | 53.1 | 94.6 | 77.9 | 58.6 | 96.4 |
| 0-I | 24.0 | 18.1 | 27.8 | 23.4 | 20.0 | 27.7 |
| II | 20.9 | 24.4 | 28.0 | 21.2 | 24.3 | 27.9 |
| III | 18.4 | 17.3 | 14.8 | 18.9 | 18.6 | 15.4 |
| IV | 36.7 | 40.2 | 29.4 | 36.5 | 37.1 | 29.0 |
| **2014-16** | | | | | | |
| No info | 35.0 | 39.8 | 1.8 | 35.0 | 39.8 | 1.8 |
| Partly | 12.9 | 5.6 | 1.5 | 3.2 | 1.9 | 0.9 |
| TNM-inf | 52.1 | 54.6 | 96.6 | 61.8 | 58.3 | 97.2 |
| 0-I | 26.5 | 22.0 | 28.3 | 26.9 | 23.0 | 28.1 |
| II | 29.2 | 26.3 | 26.7 | 29.1 | 25.4 | 26.8 |
| III | 10.6 | 18.6 | 14.0 | 11.2 | 19.8 | 13.9 |
| IV | 33.6 | 33.1 | 31.1 | 32.8 | 31.7 | 31.2 |

Supplementary Table 6. Oropharynx cancer. Time trends for TNM stages in Denmark, Norway, and Sweden over 2004-2008, 2009-2013 and 2014-2016

| **Oropharynx** | Official TNM | | | Using N0M0 for NXMX | | |
|---|---|---|---|---|---|---|
| | Denmark | Norway | Sweden | Denmark | Norway | Sweden |
| **2004-09** | | | | | | |
| No info | 7.8 | 21.0 | 10.1 | 7.8 | 21.0 | 10.1 |
| Partly | 18.1 | 5.5 | 11.0 | 2.8 | 1.0 | 0.9 |
| TNM-inf | 74.1 | 73.5 | 78.9 | 89.4 | 78.0 | 89.0 |
| 0-I | 5.3 | 6.3 | 4.5 | 6.3 | 7.0 | 6.2 |
| II | 12.5 | 11.4 | 8.5 | 13.3 | 11.4 | 10.4 |
| III | 21.2 | 15.3 | 23.3 | 22.5 | 15.3 | 23.1 |
| IV | 61.0 | 67.1 | 63.6 | 57.9 | 66.3 | 60.3 |
| **2009-13** | | | | | | |
| No info | 13.1 | 17.9 | 0.7 | 13.1 | 17.9 | 0.7 |
| Partly | 13.7 | 8.1 | 1.5 | 3.0 | 1.9 | 0.2 |
| TNM-inf | 73.2 | 74.1 | 97.8 | 83.9 | 80.3 | 99.1 |
| 0-I | 5.2 | 4.7 | 3.2 | 5.9 | 4.6 | 3.3 |
| II | 11.9 | 8.4 | 11.3 | 12.9 | 8.5 | 11.6 |
| III | 19.1 | 15.9 | 17.3 | 19.4 | 16.5 | 17.2 |
| IV | 63.7 | 71.0 | 68.2 | 61.8 | 70.3 | 67.9 |
| **2014-16** | | | | | | |
| No info | 30.8 | 21.0 | 0.6 | 30.8 | 21.0 | 0.6 |
| Partly | 9.6 | 5.1 | 0.5 | 2.5 | 1.4 | 0.3 |
| TNM-inf | 59.7 | 73.9 | 98.9 | 66.7 | 77.7 | 99.1 |
| 0-I | 6.3 | 2.1 | 3.5 | 6.9 | 2.0 | 3.5 |
| II | 11.4 | 10.1 | 9.7 | 11.8 | 10.5 | 9.7 |
| III | 17.7 | 14.9 | 13.1 | 18.0 | 15.0 | 13.0 |
| IV | 64.6 | 73.0 | 73.8 | 63.3 | 72.5 | 73.8 |

Supplementary Table 7. Nasopharynx cancer. Time trends for TNM stages in Denmark, Norway, and Sweden over 2004-2008, 2009-2013 and 2014-2016

| **Nasopharynx** | Official TNM | | | Using N0M0 for NXMX | | |
|---|---|---|---|---|---|---|
| | Denmark | Norway | Sweden | Denmark | Norway | Sweden |
| **2004-09** | | | | | | |
| No info | 8.6 | 21.3 | 13.5 | 8.6 | 21.3 | 13.5 |
| Partly | 17.2 | 8.2 | 9.6 | 4.3 | 3.3 | 4.5 |
| TNM-inf | 74.1 | 70.5 | 76.9 | 87.1 | 75.4 | 82.1 |
| 0-I | 15.1 | 9.3 | 12.5 | 12.9 | 8.7 | 12.5 |
| II | 5.8 | 0 | 7.5 | 7.9 | 2.2 | 7.8 |
| III | 12.8 | 20.9 | 16.7 | 13.9 | 19.6 | 17.2 |
| IV | 66.3 | 69.8 | 63.3 | 65.3 | 69.6 | 62.5 |
| **2009-13** | | | | | | |
| No info | 21.0 | 32.1 | 5.1 | 21.0 | 32.1 | 5.1 |
| Partly | 11.6 | 12.8 | 2.5 | 2.2 | 5.1 | 1.3 |
| TNM-inf | 67.4 | 55.1 | 92.4 | 76.8 | 62.8 | 93.6 |
| 0-I | 7.5 | 2.3 | 7.6 | 6.6 | 2.0 | 7.5 |
| II | 9.7 | 7.0 | 6.2 | 10.4 | 6.1 | 6.1 |
| III | 8.6 | 25.6 | 26.9 | 10.4 | 28.6 | 27.2 |
| IV | 74.2 | 65.1 | 59.3 | 72.6 | 63.3 | 59.2 |
| **2014-16** | | | | | | |
| No info | 34.5 | 23.8 | 1.0 | 34.5 | 23.8 | 1.0 |
| Partly | 8.6 | 9.5 | 3.0 | 1.7 | 0 | 2.0 |
| TNM-inf | 56.9 | 66.7 | 96.0 | 63.8 | 76.2 | 97.0 |
| 0-I | 9.1 | 3.6 | 8.4 | 10.8 | 6.2 | 9.4 |
| II | 9.1 | 3.6 | 4.2 | 10.8 | 6.2 | 4.2 |
| III | 27.3 | 25.0 | 28.4 | 24.3 | 21.9 | 28.1 |
| IV | 54.5 | 67.9 | 58.9 | 54.1 | 65.6 | 58.3 |

Supplementary Table 8. Hypopharynx cancer. Time trends for TNM stages in Denmark, Norway, and Sweden over 2004-2008, 2009-2013 and 2014-2016

| **Hypopharynx** | Official TNM | | | Using N0M0 for NXMX | | |
|---|---|---|---|---|---|---|
| | Denmark | Norway | Sweden | Denmark | Norway | Sweden |
| **2004-09** | | | | | | |
| No info | 7.1 | 26.4 | 13.1 | 7.1 | 26.4 | 13.1 |
| Partly | 19.1 | 13.6 | 12.4 | 2.8 | 2.1 | 1.0 |
| TNM-inf | 73.8 | 60.0 | 74.5 | 90.1 | 71.4 | 86.0 |
| 0-I | 6.7 | 8.3 | 3.4 | 8.6 | 8.0 | 4.4 |
| II | 11.8 | 10.7 | 9.8 | 11.8 | 12.0 | 10.7 |
| III | 24.0 | 15.5 | 23.1 | 22.0 | 15.0 | 25.2 |
| IV | 57.5 | 65.5 | 63.7 | 57.6 | 65.0 | 59.6 |
| **2009-13** | | | | | | |
| No info | 15.4 | 26.9 | 2.5 | 15.4 | 26.9 | 2.5 |
| Partly | 16.4 | 13.0 | 2.5 | 2.1 | 0 | 0 |
| TNM-inf | 68.2 | 60.2 | 95.1 | 82.4 | 73.1 | 97.5 |
| 0-I | 4.3 | 1.5 | 3.7 | 4.7 | 1.3 | 3.6 |
| II | 9.5 | 6.2 | 11.9 | 10.7 | 6.3 | 12.0 |
| III | 22.1 | 10.8 | 17.5 | 21.8 | 12.7 | 17.4 |
| IV | 64.2 | 81.5 | 66.9 | 62.8 | 79.7 | 67.0 |
| **2014-16** | | | | | | |
| No info | 26.5 | 21.2 | 1.2 | 26.5 | 21.2 | 1.2 |
| Partly | 10.9 | 7.1 | 0 | 0 | 1.2 | 0 |
| TNM-inf | 62.6 | 71.8 | 98.8 | 73.5 | 77.6 | 98.8 |
| 0-I | 6.6 | 0 | 3.0 | 6.5 | 0 | 3.0 |
| II | 10.2 | 8.2 | 4.8 | 10.4 | 9.1 | 4.8 |
| III | 16.8 | 18.0 | 16.3 | 20.9 | 16.7 | 16.3 |
| IV | 66.3 | 73.8 | 75.9 | 62.2 | 74.2 | 75.9 |

Supplementary Table 9. Oesophagus cancer. Time trends for TNM stages in Denmark, Norway, and Sweden over 2004-2008, 2009-2013 and 2014-2016

| **Oesophagus** | Official TNM | | | Using N0M0 for NXMX | | |
|---|---|---|---|---|---|---|
| | Denmark | Norway | Sweden | Denmark | Norway | Sweden |
| **2004-09** | | | | | | |
| No info | 14.2 | 54.7 | 26.4 | 14.2 | 54.7 | 26.4 |
| Partly | 12.0 | 14.3 | 14.9 | 4.4 | 8.5 | 5.6 |
| TNM-inf | 73.8 | 31.0 | 58.6 | 81.4 | 36.8 | 68.0 |
| 0-I | 6.8 | 18.0 | 15.5 | 7.9 | 17.7 | 17.2 |
| II | 8.6 | 15.4 | 17.4 | 9.6 | 18.2 | 19.1 |
| III | 37.7 | 27.2 | 29.4 | 40.0 | 30.9 | 31.2 |
| IV | 46.9 | 39.3 | 37.7 | 42.6 | 33.1 | 32.6 |
| **2009-13** | | | | | | |
| No info | 11.0 | 49.6 | 6.5 | 11.0 | 49.6 | 6.5 |
| Partly | 10.7 | 11.0 | 14.8 | 4.7 | 5.4 | 9.1 |
| TNM-inf | 78.3 | 39.4 | 78.9 | 84.3 | 45.1 | 84.4 |
| 0-I | 7.6 | 16.6 | 13.6 | 8.5 | 17.1 | 14.6 |
| II | 11.4 | 13.2 | 17.8 | 11.9 | 15.3 | 19.0 |
| III | 35.8 | 31.3 | 29.5 | 37.7 | 33.5 | 29.9 |
| IV | 45.2 | 38.9 | 39.1 | 42.0 | 34.1 | 36.5 |
| **2014-16** | | | | | | |
| No info | 22.0 | 54.8 | 6.6 | 22.0 | 54.8 | 6.6 |
| Partly | 11.3 | 6.7 | 6.9 | 4.5 | 3.8 | 5.1 |
| TNM-inf | 66.7 | 38.5 | 86.5 | 73.5 | 41.4 | 88.3 |
| 0-I | 8.8 | 17.7 | 12.6 | 9.1 | 18.7 | 13.3 |
| II | 12.3 | 16.2 | 17.0 | 13.3 | 17.8 | 17.3 |
| III | 39.6 | 32.6 | 33.3 | 41.9 | 32.3 | 33.1 |
| IV | 39.4 | 33.5 | 37.1 | 35.7 | 31.2 | 36.3 |

Supplementary Table 10. Stomach cancer. Time trends for TNM stages in Denmark, Norway, and Sweden over 2004-2008, 2009-2013 and 2014-2016

| **Stomach** | Official TNM | | | Using N0M0 for NXMX | | |
|---|---|---|---|---|---|---|
| | Denmark | Norway | Sweden | Denmark | Norway | Sweden |
| **2004-09** | | | | | | |
| No info | 12.6 | 59.3 | 26.4 | 12.6 | 59.3 | 26.4 |
| Partly | 10.7 | 11.1 | 18,8 | 3.6 | 2.4 | 5.9 |
| TNM-inf | 76.8 | 29.7 | 54.8 | 83.9 | 38.3 | 67.7 |
| 0-I | 9.3 | 25.9 | 20.3 | 10.9 | 27.0 | 22.1 |
| II | 14.5 | 23.3 | 21.7 | 16.9 | 29.0 | 26.5 |
| III | 22.4 | 11.1 | 13.0 | 22.9 | 13.3 | 14.9 |
| IV | 53.8 | 39.7 | 45.1 | 49.3 | 30.7 | 36.5 |
| **2009-13** | | | | | | |
| No info | 11.5 | 49.8 | 10.9 | 11.5 | 49.8 | 10.9 |
| Partly | 10.6 | 11.2 | 16.4 | 4.7 | 3.5 | 9.4 |
| TNM-inf | 77.9 | 39.0 | 72.7 | 83.8 | 46.8 | 79.6 |
| 0-I | 10.1 | 26.0 | 18.1 | 11.3 | 26.1 | 19.2 |
| II | 16.3 | 21.1 | 20.8 | 18.1 | 25.8 | 23.7 |
| III | 24.7 | 15.7 | 14.6 | 25.1 | 17.1 | 14.6 |
| IV | 48.9 | 37.2 | 46.5 | 45.5 | 31.0 | 42.4 |
| **2014-16** | | | | | | |
| No info | 18.9 | 52.9 | 11.2 | 18.9 | 52.9 | 11.2 |
| Partly | 11.2 | 8.1 | 8.3 | 4.0 | 2.8 | 4.4 |
| TNM-inf | 69.8 | 39.0 | 80.5 | 77.1 | 44.3 | 84.4 |
| 0-I | 15.9 | 19.4 | 16.3 | 16.8 | 21.4 | 17.5 |
| II | 15.4 | 20.7 | 23.3 | 18.0 | 22.6 | 24.2 |
| III | 28.1 | 26.2 | 17.0 | 28.3 | 26.3 | 17.0 |
| IV | 40.6 | 33.8 | 43.3 | 36.8 | 29.7 | 41.3 |

Supplementary Table 11. Small intestine cancer. Time trends for TNM stages in Denmark, Norway, and Sweden over 2004-2008, 2009-2013 and 2014-2016

| **Small intestine** | Official TNM | | | Using N0M0 for NXMX | | |
|---|---|---|---|---|---|---|
| | Denmark | Norway | Sweden | Denmark | Norway | Sweden |
| **2004-09** | | | | | | |
| No info | 13.8 | 62.5 | 46.2 | 13.8 | 62.5 | 46.2 |
| Partly | 18.2 | 9.4 | 14.4 | 4.0 | 1.7 | 1.4 |
| TNM-inf | 68.0 | 28.1 | 39.4 | 82.2 | 35.8 | 52.4 |
| 0-I | 8.6 | 14.0 | 15.6 | 10.8 | 15.7 | 15.5 |
| II | 14.1 | 13.3 | 18.0 | 21.3 | 19.9 | 24.2 |
| III | 27.5 | 30.7 | 22.1 | 26.7 | 31.4 | 26.9 |
| IV | 49.8 | 42.0 | 44.3 | 41.2 | 33.0 | 33.3 |
| **2009-13** | | | | | | |
| No info | 16.0 | 57.0 | 33.9 | 16.0 | 57.0 | 33.9 |
| Partly | 19.6 | 8.5 | 22.1 | 7.2 | 1.5 | 2.6 |
| TNM-inf | 64.4 | 34.4 | 44.0 | 76.8 | 41.5 | 63.5 |
| 0-I | 7.3 | 15.0 | 12.1 | 8.8 | 15.7 | 12.6 |
| II | 13.7 | 14.2 | 11.6 | 18.6 | 17.6 | 17.6 |
| III | 19.5 | 28.0 | 30.1 | 22.7 | 31.0 | 37.7 |
| IV | 59.5 | 42.9 | 46.3 | 49.9 | 35.6 | 32.1 |
| **2014-16** | | | | | | |
| No info | 19.9 | 52.9 | 30.7 | 19.9 | 52.9 | 30.7 |
| Partly | 15.9 | 23.5 | 20.9 | 4.4 | 0.2 | 2.2 |
| TNM-inf | 64.2 | 23.7 | 48.4 | 75.7 | 46.9 | 67.1 |
| 0-I | 8.9 | 10.5 | 11.1 | 11.1 | 10.6 | 11.5 |
| II | 9.3 | 12.1 | 14.6 | 12.2 | 16.3 | 18.5 |
| III | 25.4 | 33.9 | 25.7 | 28.9 | 51.2 | 34.9 |
| IV | 56.4 | 43.5 | 48.6 | 47.8 | 22.0 | 35.1 |

Supplementary Table 12. Colon cancer. Time trends for TNM stages in Denmark, Norway, Sweden, and Iceland over 2004-2008, 2009-2013 and 2014-2016

| **Colon** | Official TNM | | | | Using N0M0 for NXMX | | | |
|---|---|---|---|---|---|---|---|---|
| | Denmark | Norway | Sweden | Iceland | Denmark | Norway | Sweden | Iceland |
| **2004-09** | | | | | | | | |
| No info | 7.3 | 36.3 | 12.0 | 5.9 | 7.3 | 36.3 | 12.0 | 5.9 |
| Partly | 13.7 | 17.0 | 19.0 | 6.5 | 3.6 | 2.7 | 1.2 | 1.0 |
| TNM-inf | 78.9 | 46.7 | 69.0 | 87.6 | 89.1 | 61.0 | 86.8 | 93.1 |
| 0-I | 10.4 | 15.9 | 12.9 | 12.8 | 11.6 | 16.3 | 14.7 | 15.0 |
| II | 30.7 | 34.6 | 33.2 | 34.2 | 33.0 | 37.2 | 34.8 | 34.1 |
| III | 26.7 | 23.3 | 28.2 | 25.9 | 26.8 | 26.4 | 30.0 | 25.4 |
| IV | 32.2 | 26.2 | 25.7 | 27.0 | 28.6 | 20.0 | 20.5 | 25.4 |
| **2009-13** | | | | | | | | |
| No info | 8.8 | 17.5 | 3.1 | 6.9 | 8.8 | 17.5 | 3.1 | 6.9 |
| Partly | 13.0 | 62.9 | 12.6 | 10.8 | 5.9 | 1.5 | 2.2 | 1.4 |
| TNM-inf | 78.2 | 19.6 | 84.3 | 82.3 | 85.3 | 81.0 | 94.7 | 91.7 |
| 0-I | 9.9 | 12.2 | 13.4 | 15.3 | 11.3 | 14.8 | 15.4 | 18.4 |
| II | 31.0 | 31.5 | 32.0 | 27.7 | 32.6 | 40.9 | 32.8 | 29.3 |
| III | 26.5 | 22.5 | 30.0 | 27.7 | 26.1 | 36.1 | 30.0 | 25.9 |
| IV | 32.6 | 33.7 | 24.6 | 29.4 | 29.9 | 8.2 | 21.9 | 26.3 |
| **2014-16** | | | | | | | | |
| No info | 12.3 | 13.5 | 5.7 | 7.1 | 12.3 | 13.5 | 5.7 | 7.1 |
| Partly | 18.1 | 44.8 | 5.0 | 12.2 | 7.2 | 1.4 | 1.4 | 3.4 |
| TNM-inf | 69.6 | 41.7 | 89.3 | 80.7 | 80.5 | 85.1 | 92.9 | 89.4 |
| 0-I | 19.1 | 14.3 | 15.0 | 16.1 | 21.2 | 18.3 | 16.9 | 20.1 |
| II | 29.6 | 30.5 | 31.9 | 25.9 | 31.2 | 35.9 | 31.6 | 24.9 |
| III | 25.6 | 21.7 | 31.8 | 26.2 | 25.4 | 29.4 | 31.0 | 26.3 |
| IV | 25.7 | 33.5 | 21.3 | 31.8 | 22.2 | 16.4 | 20.4 | 28.7 |

Supplementary Table 13. Rectum cancer. Time trends for TNM stages in Denmark, Norway, Sweden, and Iceland over 2004-2008, 2009-2013 and 2014-2016

| **Rectum** | Official TNM | | | | Using N0M0 for NXMX | | | |
|---|---|---|---|---|---|---|---|---|
| | Denmark | Norway | Sweden | Iceland | Denmark | Norway | Sweden | Iceland |
| **2004-09** | | | | | | | | |
| No info | 8.5 | 33.8 | 16.1 | 100.0 | 8.5 | 33.8 | 16.1 | 100.0 |
| Partly | 16.0 | 19.3 | 15.4 | | 3.6 | 2.5 | 2.3 | |
| TNM-inf | 75.4 | 46.9 | 68.4 | | 87.8 | 63.7 | 81.5 | |
| 0-I | 13.7 | 23.6 | 22.8 | | 15.6 | 25.4 | 26.4 | |
| II | 23.8 | 24.3 | 24.2 | | 28.5 | 29.9 | 25.3 | |
| III | 35.8 | 28.5 | 29.6 | | 33.0 | 27.4 | 28.6 | |
| IV | 26.7 | 23.6 | 23.5 | | 22.9 | 17.3 | 19.7 | |
| **2009-13** | | | | | | | | |
| No info | 9.4 | 30.7 | 5.1 | 26.3 | 9.4 | 30.7 | 5.1 | 26.3 |
| Partly | 14.9 | 49.9 | 13.1 | 12.4 | 5.4 | 2.3 | 3.3 | 3.2 |
| TNM-inf | 75.6 | 19.5 | 81.9 | 61.3 | 85.1 | 67.1 | 91.6 | 70.4 |
| 0-I | 14.8 | 24.5 | 23.8 | 32.5 | 17.2 | 29.9 | 26.9 | 38.2 |
| II | 22.4 | 22.3 | 23.0 | 21.1 | 25.2 | 30.3 | 23.6 | 19.8 |
| III | 35.7 | 26.6 | 29.3 | 36.8 | 33.6 | 32.1 | 28.2 | 33.6 |
| IV | 27.0 | 26.6 | 23.8 | 9.6 | 24.0 | 7.7 | 21.3 | 8.4 |
| **2014-16** | | | | | | | | |
| No info | 14.0 | 23.4 | 8.2 | 6.1 | 14.0 | 23.4 | 8.2 | 6.1 |
| Partly | 20.7 | 33.0 | 7.1 | 15.0 | 6.6 | 1.5 | 1.8 | 5.4 |
| TNM-inf | 65.3 | 43.7 | 84.7 | 78.9 | 79.4 | 75.1 | 90.0 | 88.4 |
| 0-I | 25.0 | 25.5 | 26.9 | 25.9 | 27.7 | 29.2 | 29.9 | 30.8 |
| II | 21.7 | 21.2 | 22.1 | 25.0 | 25.1 | 26.6 | 21.7 | 23.8 |
| III | 31.5 | 27.9 | 31.4 | 31.9 | 29.2 | 29.4 | 29.9 | 30.0 |
| IV | 21.9 | 25.4 | 19.6 | 17.2 | 18.0 | 14.8 | 18.5 | 15.4 |

Supplementary Table 14. Anal cancer. Time trends for TNM stages in Denmark, Norway, and Sweden over 2004-2008, 2009-2013 and 2014-2016

| **Anus** | Official TNM | | | Using N0M0 for NXMX | | |
|---|---|---|---|---|---|---|
| | Denmark | Norway | Sweden | Denmark | Norway | Sweden |
| **2004-09** | | | | | | |
| No info | 22.1 | 45.0 | 54.5 | 22.1 | 45.0 | 54.5 |
| Partly | 21.7 | 12.5 | 21.9 | 5.0 | 3.5 | 5.2 |
| TNM-inf | 56.2 | 42.5 | 23.7 | 72.9 | 51.4 | 40.3 |
| 0-I | 12.9 | 11.3 | 28.9 | 13.8 | 11.2 | 27.3 |
| II | 39.3 | 32.3 | 35.8 | 41.9 | 34.8 | 42.4 |
| III | 36.1 | 42.9 | 26.4 | 35.3 | 42.9 | 25.1 |
| IV | 11.8 | 13.5 | 8.8 | 9.1 | 11.2 | 5.2 |
| **2009-13** | | | | | | |
| No info | 19.7 | 48.6 | 57.0 | 19.7 | 48.6 | 57.0 |
| Partly | 18.0 | 12.2 | 14.6 | 4.7 | 1.4 | 3.4 |
| TNM-inf | 62.3 | 39.2 | 28.3 | 75.6 | 50.0 | 39.6 |
| 0-I | 9.2 | 10.1 | 18.4 | 10.0 | 9.7 | 20.5 |
| II | 38.6 | 39.1 | 30.0 | 41.6 | 35.8 | 32.0 |
| III | 37.4 | 39.1 | 36.4 | 36.2 | 45.5 | 36.6 |
| IV | 14.8 | 11.6 | 15.2 | 12.2 | 9.1 | 10.9 |
| **2014-16** | | | | | | |
| No info | 47.8 | 67.0 | 46.8 | 47.8 | 67.0 | 46.8 |
| Partly | 32.3 | 8.1 | 10.0 | 11.2 | 1.9 | 1.5 |
| TNM-inf | 19.9 | 24.8 | 43.2 | 41.0 | 31.1 | 51.6 |
| 0-I | 17.5 | 7.5 | 11.6 | 13.3 | 9.5 | 16.4 |
| II | 25.0 | 40.3 | 29.8 | 37.0 | 44.0 | 30.5 |
| III | 40.0 | 38.8 | 42.7 | 41.2 | 35.7 | 39.8 |
| IV | 17.5 | 13.4 | 16.0 | 8.5 | 10.7 | 13.4 |

Supplementary Table 15. Liver cancer. Time trends for TNM stages in Denmark, Norway, and Sweden over 2004-2008, 2009-2013 and 2014-2016

| **Liver** | Official TNM | | | Using N0M0 for NXMX | | |
|---|---|---|---|---|---|---|
| | Denmark | Norway | Sweden | Denmark | Norway | Sweden |
| **2004-09** | | | | | | |
| No info | 26.1 | 76.3 | 53.2 | 26.1 | 76.3 | 53.2 |
| Partly | 24.9 | 7.9 | 15.2 | 8.2 | 2.1 | 2.1 |
| TNM-inf | 49.0 | 15.8 | 31.6 | 65.7 | 21.6 | 44.7 |
| 0-I | 9.6 | 8.8 | 18.5 | 10.4 | 7.1 | 17.6 |
| II | 8.9 | 14.2 | 14.1 | 10.5 | 15.6 | 14.6 |
| III | 22.4 | 28.3 | 16.3 | 32.7 | 35.7 | 26.9 |
| IV | 59.2 | 48.7 | 51.2 | 46.4 | 41.6 | 41.0 |
| **2009-13** | | | | | | |
| No info | 22.7 | 70.4 | 20.2 | 22.7 | 70.4 | 20.2 |
| Partly | 20.6 | 8.1 | 25.9 | 8.5 | 2.0 | 8.6 |
| TNM-inf | 56.7 | 21.4 | 53.8 | 68.8 | 27.6 | 71.2 |
| 0-I | 11.2 | 12.3 | 21.9 | 13.3 | 11.6 | 23.9 |
| II | 12.4 | 15.4 | 19.1 | 12.9 | 17.8 | 19.9 |
| III | 26.7 | 23.3 | 17.6 | 30.9 | 29.1 | 22.7 |
| IV | 49.7 | 48.9 | 41.4 | 42.9 | 41.4 | 33.5 |
| **2014-16** | | | | | | |
| No info | 28.7 | 65.3 | 11.0 | 28.7 | 65.3 | 11.0 |
| Partly | 16.5 | 18.5 | 12.2 | 8.1 | 0.8 | 8.2 |
| TNM-inf | 54.8 | 16.2 | 76.8 | 63.3 | 33.9 | 80.8 |
| 0-I | 17.4 | 7.9 | 28.5 | 18.4 | 19.7 | 28.8 |
| II | 16.2 | 15.1 | 21.2 | 16.9 | 27.3 | 21.3 |
| III | 24.5 | 21.4 | 15.8 | 26.3 | 20.8 | 16.7 |
| IV | 41.8 | 55.6 | 34.5 | 38.4 | 32.2 | 33.3 |

Supplementary Table 16. Gallbladder cancer. Time trends for TNM stages in Denmark, Norway, Sweden, and Iceland over 2004-2008, 2009-2013 and 2014-2016

| **Gallbladder** | Official TNM | | | Using N0M0 for NXMX | | |
|---|---|---|---|---|---|---|
| | Denmark | Norway | Sweden | Denmark | Norway | Sweden |
| **2004-09** | | | | | | |
| No info | 24.1 | 63.1 | 32.3 | 24.1 | 63.1 | 32.3 |
| Partly | 20.7 | 10.5 | 21.0 | 8.3 | 2.5 | 2.3 |
| TNM-inf | 55.2 | 26.4 | 46.7 | 67.5 | 34.4 | 65.4 |
| 0-I | 14.1 | 25.1 | 16.3 | 18.5 | 25.5 | 18.0 |
| II | 14.7 | 19.3 | 16.0 | 17.0 | 23.9 | 21.0 |
| III | 10.9 | 9.6 | 13.6 | 13.3 | 13.2 | 19.2 |
| IV | 60.3 | 46.0 | 54.1 | 51.2 | 37.4 | 41.8 |
| **2009-13** | | | | | | |
| No info | 17.4 | 56.1 | 16.8 | 17.4 | 56.1 | 16.8 |
| Partly | 18.7 | 10.1 | 27.6 | 7.7 | 3.9 | 8.9 |
| TNM-inf | 63.9 | 33.8 | 55.6 | 74.9 | 40.1 | 74.2 |
| 0-I | 11.6 | 17.7 | 14.1 | 15.1 | 18.2 | 16.9 |
| II | 17.0 | 28.5 | 19.0 | 18.8 | 31.4 | 24.5 |
| III | 9.8 | 9.7 | 15.8 | 12.0 | 12.3 | 18.3 |
| IV | 61.5 | 44.1 | 51.1 | 54.2 | 38.1 | 40.2 |
| **2014-16** | | | | | | |
| No info | 23.2 | 52.0 | 16.6 | 23.2 | 52.0 | 16.6 |
| Partly | 19.1 | 22.2 | 16.3 | 8.9 | 1.4 | 6.5 |
| TNM-inf | 57.7 | 25.7 | 67.1 | 67.9 | 46.5 | 76.9 |
| 0-I | 10.3 | 14.3 | 16.4 | 12.7 | 20.6 | 16.5 |
| II | 24.4 | 39.7 | 21.2 | 28.6 | 45.2 | 25.3 |
| III | 10.6 | 12.7 | 15.1 | 11.5 | 14.9 | 16.9 |
| IV | 54.7 | 33.3 | 47.3 | 47.2 | 19.3 | 41.4 |

Supplementary Table 17. Pancreas cancer. Time trends for TNM stages in Denmark, Norway, and Sweden over 2004-2008, 2009-2013 and 2014-2016

| **Pancreas** | Official TNM | | | Using N0M0 for NXMX | | |
|---|---|---|---|---|---|---|
| | Denmark | Norway | Sweden | Denmark | Norway | Sweden |
| **2004-09** | | | | | | |
| No info | 16.1 | 66.1 | 34.4 | 16.1 | 66.1 | 34.4 |
| Partly | 12.7 | 8.3 | 15.0 | 4.2 | 0.9 | 1.1 |
| TNM-inf | 71.2 | 25.6 | 50.6 | 79.7 | 33.0 | 64.5 |
| 0-I | 5.9 | 12.3 | 11.0 | 8.9 | 15.6 | 13.1 |
| II | 10.4 | 14.8 | 16.1 | 13.1 | 20.5 | 22.7 |
| III | 15.8 | 8.4 | 9.4 | 17.2 | 13.8 | 14.3 |
| IV | 67.9 | 64.5 | 63.5 | 60.7 | 50.0 | 49.8 |
| **2009-13** | | | | | | |
| No info | 15.0 | 58.0 | 15.8 | 15.0 | 58.0 | 15.8 |
| Partly | 10.9 | 6.6 | 21.0 | 4.9 | 1.1 | 3.0 |
| TNM-inf | 74.1 | 35.4 | 63.2 | 80.1 | 40.9 | 81.2 |
| 0-I | 4.8 | 9.1 | 6.4 | 6.7 | 10.4 | 9.9 |
| II | 15.1 | 15.8 | 18.5 | 16.4 | 19.2 | 25.1 |
| III | 12.2 | 9.9 | 12.2 | 14.1 | 13.9 | 16.1 |
| IV | 67.9 | 65.2 | 62.8 | 62.8 | 56.5 | 48.9 |
| **2014-16** | | | | | | |
| No info | 22.3 | 62.9 | 14.4 | 22.3 | 62.9 | 14.4 |
| Partly | 10.6 | 10.8 | 16.9 | 4.7 | 0.6 | 1.9 |
| TNM-inf | 67.1 | 26.3 | 68.6 | 73.0 | 36.5 | 83.6 |
| 0-I | 5.1 | 10.2 | 4.7 | 7.0 | 14.2 | 7.3 |
| II | 20.7 | 22.3 | 19.5 | 22.6 | 34.0 | 26.3 |
| III | 9.8 | 11.0 | 12.4 | 11.2 | 11.1 | 14.4 |
| IV | 64.4 | 56.6 | 63.4 | 59.2 | 40.7 | 52.0 |

Supplementary Table 18. Nose, Sinus cancer. Time trends for TNM stages in Denmark, Norway, and Sweden over 2004-2008, 2009-2013 and 2014-2016

| **Nose, sinus** | Official TNM | | | Using N0M0 for NXMX | | |
|---|---|---|---|---|---|---|
| | Denmark | Norway | Sweden | Denmark | Norway | Sweden |
| **2004-09** | | | | | | |
| No info | 10.3 | 28.9 | 20.3 | 10.3 | 28.9 | 20.3 |
| Partly | 15.6 | 10.1 | 13.9 | 5.3 | 3.9 | 2.2 |
| TNM-inf | 74.0 | 61.0 | 65.8 | 84.4 | 67.1 | 77.5 |
| 0-I | 28.7 | 17.3 | 28.7 | 28.5 | 17.0 | 26.9 |
| II | 20.4 | 16.5 | 10.5 | 19.9 | 15.7 | 11.1 |
| III | 14.7 | 10.8 | 16.0 | 14.9 | 11.1 | 15.8 |
| IV | 36.2 | 55.4 | 44.7 | 36.8 | 56.2 | 46.2 |
| **2009-13** | | | | | | |
| No info | 17.3 | 47.7 | 8.8 | 17.3 | 47.7 | 8.8 |
| Partly | 14.4 | 7.8 | 1.7 | 3.5 | 2.3 | 0.0 |
| TNM-inf | 68.3 | 44.5 | 89.5 | 79.2 | 50.0 | 91.2 |
| 0-I | 32.2 | 13.4 | 16.5 | 32.2 | 14.7 | 16.2 |
| II | 18.0 | 16.5 | 14.3 | 18.5 | 15.6 | 15.0 |
| III | 6.4 | 6.2 | 15.6 | 7.0 | 7.3 | 15.6 |
| IV | 43.3 | 63.9 | 53.7 | 42.2 | 62.4 | 53.3 |
| **2014-16** | | | | | | |
| No info | 32.7 | 51.2 | 10.1 | 32.7 | 51.2 | 10.1 |
| Partly | 10.9 | 3.1 | 0.5 | 2.5 | 1.6 | 0.5 |
| TNM-inf | 56.4 | 45.7 | 89.4 | 64.9 | 47.3 | 89.4 |
| 0-I | 31.6 | 16.9 | 19.8 | 32.8 | 16.4 | 19.8 |
| II | 15.8 | 6.8 | 13.6 | 14.5 | 8.2 | 13.6 |
| III | 13.2 | 10.2 | 15.3 | 13.7 | 9.8 | 15.3 |
| IV | 39.5 | 66.1 | 51.4 | 38.9 | 65.6 | 51.4 |

Supplementary Table 19. Larynx cancer. Time trends for TNM stages in Denmark, Norway, and Sweden over 2004-2008, 2009-2013 and 2014-2016

| **Larynx** | Official TNM | | | Using N0M0 for NXMX | | |
|---|---|---|---|---|---|---|
| | Denmark | Norway | Sweden | Denmark | Norway | Sweden |
| **2004-09** | | | | | | |
| No info | 4.6 | 25.4 | 9.8 | 4.6 | 25.4 | 9.8 |
| Partly | 12.8 | 6.7 | 9.6 | 1.3 | 0.3 | 0.4 |
| TNM-inf | 82.6 | 67.9 | 80.6 | 94.1 | 74.2 | 89.8 |
| 0-I | 38.2 | 32.7 | 40.4 | 38.0 | 32.2 | 38.8 |
| II | 22.0 | 26.8 | 22.1 | 22.9 | 25.5 | 22.8 |
| III | 18.1 | 16.5 | 16.2 | 18.2 | 17.8 | 17.2 |
| IV | 21.7 | 24.1 | 21.3 | 20.9 | 24.5 | 21.2 |
| **2009-13** | | | | | | |
| No info | 9.4 | 36.7 | 1.5 | 9.4 | 36.7 | 1.5 |
| Partly | 8.0 | 4.2 | 2.4 | 0.8 | 0.3 | 0.9 |
| TNM-inf | 82.6 | 59.1 | 96.1 | 89.7 | 62.9 | 97.6 |
| 0-I | 37.1 | 29.2 | 41.5 | 37.0 | 28.8 | 41.3 |
| II | 24.1 | 24.6 | 22.4 | 23.8 | 23.7 | 22.9 |
| III | 17.0 | 19.8 | 15.3 | 17.1 | 20.4 | 15.2 |
| IV | 21.9 | 26.4 | 20.8 | 22.1 | 27.2 | 20.6 |
| **2014-16** | | | | | | |
| No info | 33.3 | 35.9 | 2.5 | 33.3 | 35.9 | 2.5 |
| Partly | 7.8 | 6.1 | 0.8 | 0.8 | 0.6 | 0.4 |
| TNM-inf | 58.9 | 58.0 | 96.8 | 65.9 | 63.5 | 97.1 |
| 0-I | 39.2 | 28.5 | 47.0 | 38.7 | 27.9 | 46.8 |
| II | 24.3 | 25.0 | 17.5 | 25.4 | 25.6 | 17.6 |
| III | 13.9 | 25.0 | 15.5 | 13.7 | 24.2 | 15.7 |
| IV | 22.5 | 21.5 | 20.0 | 22.2 | 22.4 | 20.0 |

Supplementary Table 20. Lung cancer. Time trends for TNM stages in Denmark, Norway, and Sweden over 2004-2008, 2009-2013 and 2014-2016

| **Lung** | Official TNM | | | Using N0M0 for NXMX | | |
|---|---|---|---|---|---|---|
| | Denmark | Norway | Sweden | Denmark | Norway | Sweden |
| **2004-09** | | | | | | |
| No info | 5.1 | 36.4 | 7.5 | 5.1 | 36.4 | 7.5 |
| Partly | 5.6 | 5.8 | 3.9 | 0.7 | 0.4 | 0.2 |
| TNM-inf | 89.2 | 57.8 | 88.6 | 94.2 | 63.2 | 92.4 |
| 0-I | 12.2 | 20.5 | 17.9 | 13.2 | 21.2 | 18.1 |
| II | 4.8 | 5.8 | 3.7 | 5.3 | 6.3 | 4.1 |
| III | 25.0 | 25.7 | 28.3 | 26.5 | 28.5 | 29.8 |
| IV | 58.0 | 48.0 | 50.1 | 54.9 | 44.0 | 48.0 |
| **2009-13** | | | | | | |
| No info | 2.7 | 27.5 | 2.9 | 2.7 | 27.5 | 2.9 |
| Partly | 3.7 | 8.1 | 3.4 | 0.5 | 0.8 | 1.3 |
| TNM-inf | 93.6 | 64.4 | 93.7 | 96.8 | 71.6 | 95.8 |
| 0-I | 13.1 | 20.7 | 18.8 | 13.5 | 21.5 | 18.9 |
| II | 7.2 | 8.0 | 6.6 | 7.6 | 8.7 | 6.7 |
| III | 22.4 | 21.4 | 21.6 | 23.4 | 24.9 | 22.5 |
| IV | 57.3 | 49.9 | 53.0 | 55.5 | 44.8 | 51.9 |
| **2014-16** | | | | | | |
| No info | 3.3 | 18.8 | 3.4 | 3.3 | 18.8 | 3.4 |
| Partly | 3.2 | 13.8 | 0.7 | 1.0 | 3.3 | 0.2 |
| TNM-inf | 93.5 | 67.4 | 95.9 | 95.7 | 78.0 | 96.4 |
| 0-I | 17.5 | 20.6 | 19.2 | 17.6 | 24.2 | 19.3 |
| II | 8.8 | 8.5 | 7.2 | 9.0 | 10.0 | 7.4 |
| III | 21.7 | 16.6 | 19.9 | 22.6 | 18.9 | 20.0 |
| IV | 52.0 | 54.3 | 53.6 | 50.8 | 46.9 | 53.3 |

Supplementary Table 21. Pleura cancer. Time trends for TNM stages in Denmark, Norway, and Sweden over 2004-2008, 2009-2013 and 2014-2016

| Pleura | Official TNM | | | Using N0M0 for NXMX | | |
|---|---|---|---|---|---|---|
| | Denmark | Norway | Sweden | Denmark | Norway | Sweden |
| **2004-09** | | | | | | |
| No info | 8.3 | 62.1 | 48.3 | 8.3 | 62.1 | 48.3 |
| Partly | 20.2 | 8.5 | 7.2 | 4.8 | 2.6 | 1.7 |
| TNM-inf | 71.5 | 29.4 | 44.5 | 86.9 | 35.3 | 50.0 |
| 0-I | 5.5 | 18.4 | 16.2 | 6.2 | 19.7 | 16.1 |
| II | 18.3 | 21.9 | 16.2 | 22.8 | 21.9 | 16.8 |
| III | 19.1 | 15.8 | 23.0 | 20.0 | 16.8 | 22.8 |
| IV | 57.1 | 43.9 | 44.5 | 51.0 | 41.6 | 44.3 |
| **2009-13** | | | | | | |
| No info | 7.0 | 57.2 | 46.3 | 7.0 | 57.2 | 46.3 |
| Partly | 11.1 | 7.1 | 5.3 | 2.7 | 1.3 | 2.1 |
| TNM-inf | 81.8 | 35.8 | 48.4 | 90.2 | 41.6 | 51.6 |
| 0-I | 8.4 | 15.5 | 13.9 | 9.1 | 14.5 | 14.0 |
| II | 15.3 | 16.2 | 23.5 | 16.0 | 16.4 | 23.0 |
| III | 22.3 | 26.1 | 21.2 | 23.0 | 26.7 | 22.0 |
| IV | 54.0 | 42.3 | 41.4 | 51.8 | 42.4 | 41.0 |
| **2014-16** | | | | | | |
| No info | 12.9 | 60.1 | 44.1 | 12.9 | 60.1 | 44.1 |
| Partly | 12.9 | 5.4 | 7.4 | 7.1 | 1.8 | 0.3 |
| TNM-inf | 74.1 | 34.5 | 48.5 | 79.9 | 38.1 | 55.6 |
| 0-I | 13.7 | 16.9 | 15.2 | 13.7 | 17.6 | 15.3 |
| II | 18.8 | 10.4 | 27.3 | 20.0 | 11.8 | 24.9 |
| III | 19.9 | 48.1 | 18.2 | 19.4 | 47.1 | 21.2 |
| IV | 47.6 | 24.7 | 39.4 | 47.0 | 23.5 | 38.6 |

Supplementary Table 22. Breast cancer. Time trends for TNM stages in Denmark, Norway, Sweden, and Iceland over 2004-2008, 2009-2013 and 2014-2016

| Breast | Official TNM | | | | Using N0M0 for NXMX | | | |
|---|---|---|---|---|---|---|---|---|
| | Denmark | Norway | Sweden | Iceland | Denmark | Norway | Sweden | Iceland |
| **2004-09** | | | | | | | | |
| No info | 2.2 | 3.4 | 15.9 | 1.7 | 2.2 | 3.4 | 15.9 | 1.7 |
| Partly | 8.5 | 4.0 | 24.2 | 74.5 | 0.7 | 0.3 | 1.5 | 1.0 |
| TNM-inf | 89.3 | 92.6 | 59.9 | 23.9 | 97.0 | 96.3 | 82.7 | 97.4 |
| 0-I | 32.8 | 43.2 | 51.0 | 41.5 | 32.6 | 43.4 | 49.3 | 51.0 |
| II | 44.9 | 44.1 | 40.0 | 34.1 | 45.0 | 44.4 | 42.1 | 42.6 |
| III | 15.6 | 7.8 | 4.8 | 10.6 | 16.2 | 7.6 | 5.6 | 3.1 |
| IV | 6.7 | 4.8 | 4.1 | 13.8 | 6.2 | 4.7 | 3.0 | 3.4 |
| **2009-13** | | | | | | | | |
| No info | 3.8 | 4.7 | 1.5 | 2.7 | 3.8 | 4.7 | 1.5 | 2.7 |
| Partly | 8.9 | 2.7 | 12.0 | 17.8 | 0.7 | 0.2 | 2.7 | 0.5 |
| TNM-inf | 87.3 | 92.5 | 86.4 | 79.5 | 95.5 | 95.1 | 95.8 | 96.8 |
| 0-I | 46.6 | 44.3 | 58.4 | 40.9 | 45.2 | 44.1 | 56.4 | 42.4 |
| II | 38.4 | 38.9 | 34.9 | 38.0 | 39.0 | 39.5 | 36.4 | 39.2 |
| III | 10.1 | 12.8 | 3.6 | 14.7 | 11.4 | 12.5 | 4.4 | 13.2 |
| IV | 4.9 | 4.1 | 3.1 | 6.4 | 4.5 | 3.9 | 2.8 | 5.3 |
| **2014-16** | | | | | | | | |
| No info | 9.3 | 2.4 | 3.6 | 3.9 | 9.3 | 2.4 | 3.6 | 3.9 |
| Partly | 11.9 | 3.0 | 1.1 | 13.3 | 1.7 | 0.1 | 0.4 | 1.4 |
| TNM-inf | 78.8 | 94.6 | 95.3 | 82.9 | 89.0 | 97.6 | 96.0 | 94.8 |
| 0-I | 51.8 | 45.4 | 55.4 | 38.0 | 49.5 | 44.9 | 55.3 | 40.1 |
| II | 37.4 | 38.3 | 37.5 | 39.7 | 38.3 | 39.3 | 37.6 | 39.6 |
| III | 7.0 | 12.5 | 4.2 | 15.3 | 8.8 | 12.2 | 4.3 | 14.2 |
| IV | 3.8 | 3.8 | 2.8 | 7.1 | 3.3 | 3.7 | 2.8 | 6.2 |

Supplementary Table 23. Prostate cancer. Time trends for TNM stages in Denmark, Norway, Sweden, and Iceland over 2004-2008, 2009-2013 and 2014-2016

| **Prostate** | Official TNM | | | | Using N0M0 for NXMX | | | |
|---|---|---|---|---|---|---|---|---|
| | Denmark | Norway | Sweden | Iceland | Denmark | Norway | Sweden | Iceland |
| **2004-09** | | | | | | | | |
| No info | 13.5 | 10.0 | 5.5 | 4.3 | 13.5 | 10.0 | 5.5 | 4.3 |
| Partly | 45.3 | 57.3 | 80.8 | 27.2 | 1.1 | 0.9 | 0.7 | 1.1 |
| TNM-inf | 41.3 | 32.8 | 13.7 | 68.5 | 85.4 | 89.1 | 93.8 | 94.6 |
| 0-I | 18.8 | 24.1 | 15.3 | 47.9 | 31.2 | 33.8 | 44.5 | 48.8 |
| II | 21.1 | 20.7 | 16.0 | 25.2 | 25.8 | 26.4 | 29.3 | 26.8 |
| III | 17.9 | 19.0 | 10.7 | 11.2 | 20.5 | 23.7 | 15.4 | 12.7 |
| IV | 42.2 | 36.2 | 58.0 | 15.7 | 22.5 | 16.1 | 10.8 | 11.6 |
| **2009-13** | | | | | | | | |
| No info | 13.2 | 11.8 | 2.1 | 3.8 | 13.2 | 11.8 | 2.1 | 3.8 |
| Partly | 50.8 | 61.0 | 78.2 | 21.3 | 0.9 | 0.6 | 2.0 | 0.5 |
| TNM-inf | 36.0 | 27.2 | 19.7 | 74.9 | 85.9 | 87.6 | 95.9 | 95.7 |
| 0-I | 19.4 | 14.0 | 28.3 | 49.8 | 32.3 | 30.7 | 47.8 | 52.1 |
| II | 23.8 | 25.0 | 18.6 | 26.4 | 29.3 | 32.5 | 28.8 | 26.3 |
| III | 19.7 | 23.2 | 10.5 | 10.3 | 21.1 | 22.8 | 13.2 | 10.8 |
| IV | 37.2 | 37.8 | 42.6 | 13.5 | 17.3 | 14.0 | 10.2 | 10.7 |
| **2014-16** | | | | | | | | |
| No info | 19.1 | 16.0 | 1.5 | 6.8 | 19.1 | 16.0 | 1.5 | 6.8 |
| Partly | 52.6 | 39.0 | 76.3 | 15.1 | 0.5 | 0.5 | 2.0 | 0.3 |
| TNM-inf | 28.3 | 45.0 | 22.2 | 78.1 | 80.4 | 83.4 | 96.5 | 92.9 |
| 0-I | 16.5 | 15.5 | 41.0 | 38.6 | 32.8 | 22.1 | 51.6 | 40.3 |
| II | 20.1 | 38.4 | 21.8 | 28.8 | 29.3 | 40.1 | 29.0 | 27.8 |
| III | 17.3 | 25.0 | 8.7 | 16.1 | 19.9 | 24.9 | 11.7 | 15.9 |
| IV | 46.2 | 21.1 | 28.5 | 16.5 | 18.0 | 12.8 | 7.7 | 16.1 |

Supplementary Table 24. Testis cancer. Time trends for TNM stages in Denmark, Norway, and Sweden over 2004-2008, 2009-2013 and 2014-2016

| **Testis** | Official TNM | | | Using N0M0 for NXMX | | |
|---|---|---|---|---|---|---|
| | Denmark | Norway | Sweden | Denmark | Norway | Sweden |
| **2004-09** | | | | | | |
| No info | 3.7 | 45.5 | 14.6 | 3.7 | 45.5 | 14.6 |
| Partly | 16.5 | 18.5 | 22.7 | 1.2 | 1.2 | 0.8 |
| TNM-inf | 79.9 | 36.0 | 62.7 | 95.1 | 53.3 | 84.6 |
| 0-I | 71.8 | 82.6 | 75.7 | 75.5 | 86.0 | 80.0 |
| II | 11.8 | 9.4 | 14.1 | 10.7 | 8.6 | 12.5 |
| III | 16.4 | 8.0 | 10.2 | 13.8 | 5.4 | 7.6 |
| IV | 0 | 0 | 0 | 0 | 0 | 0 |
| **2009-13** | | | | | | |
| No info | 9.7 | 53.0 | 13.0 | 9.7 | 53.0 | 13.0 |
| Partly | 17.0 | 14.1 | 16.1 | 1.3 | 1.7 | 0.9 |
| TNM-inf | 73.2 | 32.8 | 70.9 | 89.0 | 45.3 | 86.1 |
| 0-I | 76.2 | 82.0 | 74.0 | 79.9 | 85.3 | 78.3 |
| II | 9.1 | 10.2 | 16.3 | 8.0 | 9.0 | 13.7 |
| III | 14.7 | 7.8 | 9.7 | 12.1 | 5.7 | 8.0 |
| IV | 0 | 0 | 0 | 0 | 0 | 0 |
| **2014-16** | | | | | | |
| No info | 15.7 | 65.1 | 6.0 | 15.7 | 65.1 | 6.0 |
| Partly | 46.3 | 8.4 | 8.1 | 3.9 | 0.8 | 0.1 |
| TNM-inf | 37.9 | 26.6 | 86.0 | 80.4 | 34.2 | 94.0 |
| 0-I | 78.4 | 86.6 | 77.4 | 88.4 | 88.9 | 79.2 |
| II | 9.6 | 8.0 | 15.8 | 5.9 | 6.9 | 14.5 |
| III | 12.0 | 5.5 | 6.8 | 5.7 | 4.2 | 6.2 |
| IV | 0 | 0 | 0 | 0 | 0 | 0 |

Supplementary Table 25. Penis etc. cancer. Time trends for TNM stages in Denmark, Norway, and Sweden over 2004-2008, 2009-2013 and 2014-2016

| **Penis etc.** | Official TNM | | | Using N0M0 for NXMX | | |
|---|---|---|---|---|---|---|
| | Denmark | Norway | Sweden | Denmark | Norway | Sweden |
| **2004-09** | | | | | | |
| No info | 11.7 | 37.4 | 10.3 | 11.7 | 37.4 | 10.3 |
| Partly | 42.1 | 27.0 | 47.4 | 6.8 | 12.6 | 4.9 |
| TNM-inf | 46.2 | 35.6 | 42.3 | 81.6 | 50.0 | 84.8 |
| 0-I | 46.3 | 38.0 | 28.2 | 48.4 | 35.1 | 40.2 |
| II | 26.8 | 34.2 | 38.7 | 32.3 | 41.4 | 38.6 |
| III | 13.0 | 22.8 | 21.0 | 11.1 | 19.8 | 13.8 |
| IV | 13.8 | 5.1 | 12.2 | 8.3 | 3.6 | 7.4 |
| **2009-13** | | | | | | |
| No info | 17.5 | 37.4 | 5.5 | 17.5 | 37.4 | 5.5 |
| Partly | 40.0 | 24.3 | 36.6 | 7.8 | 7.2 | 6.5 |
| TNM-inf | 42.5 | 38.3 | 58.0 | 74.7 | 55.4 | 88.1 |
| 0-I | 36.0 | 36.5 | 39.0 | 42.7 | 37.4 | 45.2 |
| II | 28.7 | 31.8 | 27.2 | 33.1 | 38.2 | 29.8 |
| III | 16.9 | 18.8 | 19.5 | 13.4 | 15.4 | 14.9 |
| IV | 18.4 | 12.9 | 14.3 | 10.9 | 8.9 | 10.1 |
| **2014-16** | | | | | | |
| No info | 22.8 | 68.0 | 9.0 | 22.8 | 68.0 | 9.0 |
| Partly | 39.7 | 13.0 | 29.0 | 7.3 | 4.0 | 9.6 |
| TNM-inf | 37.4 | 19.0 | 62.0 | 69.9 | 28.0 | 81.4 |
| 0-I | 36.6 | 39.5 | 32.4 | 43.1 | 46.4 | 39.7 |
| II | 30.5 | 31.6 | 38.2 | 34.0 | 32.1 | 36.4 |
| III | 19.5 | 15.8 | 20.8 | 15.0 | 10.7 | 16.9 |
| IV | 13.4 | 13.2 | 8.7 | 7.8 | 10.7 | 7.0 |

Supplementary Table 26. Kidney cancer. Time trends for TNM stages in Denmark, Norway, and Sweden over 2004-2008, 2009-2013 and 2014-2016

| **Kidney** | Official TNM | | | Using N0M0 for NXMX | | |
|---|---|---|---|---|---|---|
| | Denmark | Norway | Sweden | Denmark | Norway | Sweden |
| **2004-09** | | | | | | |
| No info | 10.7 | 35.8 | 12.2 | 10.7 | 35.8 | 12.2 |
| Partly | 16.3 | 20.7 | 36.1 | 1.7 | 1.2 | 0.6 |
| TNM-inf | 73.0 | 43.5 | 51.7 | 87.6 | 63.0 | 87.2 |
| 0-I | 32.8 | 42.8 | 34.9 | 34.7 | 44.3 | 43.8 |
| II | 13.6 | 20.1 | 13.9 | 15.6 | 22.2 | 15.7 |
| III | 10.6 | 10.8 | 13.4 | 12.0 | 13.3 | 16.5 |
| IV | 43.0 | 26.3 | 37.8 | 37.7 | 20.2 | 24.0 |
| **2009-13** | | | | | | |
| No info | 14.8 | 34.6 | 4.2 | 14.8 | 34.6 | 4.2 |
| Partly | 20.4 | 15.4 | 20.1 | 1.1 | 0.8 | 0.9 |
| TNM-inf | 64.8 | 50.0 | 75.6 | 84.1 | 64.6 | 94.9 |
| 0-I | 41.7 | 56.9 | 47.0 | 45.6 | 57.2 | 50.1 |
| II | 8.9 | 12.3 | 12.7 | 10.6 | 13.1 | 13.5 |
| III | 13.9 | 9.6 | 16.2 | 15.7 | 11.6 | 16.5 |
| IV | 35.5 | 21.2 | 24.1 | 28.1 | 18.1 | 19.9 |
| **2014-16** | | | | | | |
| No info | 10.2 | 50.2 | 2.8 | 10.2 | 50.2 | 2.8 |
| Partly | 12.4 | 7.3 | 8.3 | 0.9 | 0.5 | 0.8 |
| TNM-inf | 77.4 | 42.5 | 88.8 | 88.8 | 49.3 | 96.4 |
| 0-I | 52.0 | 65.7 | 55.5 | 53.3 | 65.5 | 56.5 |
| II | 8.1 | 10.9 | 9.9 | 9.0 | 11.3 | 10.1 |
| III | 16.8 | 10.6 | 16.0 | 17.3 | 11.8 | 16.2 |
| IV | 23.1 | 12.9 | 18.6 | 20.4 | 11.3 | 17.2 |

Supplementary Table 27. Bladder cancer. Time trends for TNM stages in Denmark, Norway, and Sweden over 2004-2008, 2009-2013 and 2014-2016

| **Bladder** | Official TNM | | | Using N0M0 for NXMX | | |
|---|---|---|---|---|---|---|
| | Denmark | Norway | Sweden | Denmark | Norway | Sweden |
| **2004-09** | | | | | | |
| No info | 6.3 | 33.2 | 16.9 | 6.3 | 33.2 | 16.9 |
| Partly | 46.2 | 36.6 | 62.6 | 0.8 | 1.9 | 0.8 |
| TNM-inf | 47.5 | 30.2 | 20.5 | 92.9 | 64.9 | 82.3 |
| 0-I | 55.5 | 64.1 | 41.3 | 64.9 | 65.6 | 71.5 |
| II | 12.2 | 12.5 | 23.1 | 12.8 | 14.2 | 14.7 |
| III | 11.0 | 10.2 | 10.9 | 10.0 | 12.7 | 6.5 |
| IV | 21.3 | 13.2 | 24.7 | 12.2 | 7.5 | 7.3 |
| **2009-13** | | | | | | |
| No info | 5.7 | 47.0 | 9.2 | 5.7 | 47.0 | 9.2 |
| Partly | 45.8 | 28.5 | 62.3 | 0.8 | 7.9 | 2.3 |
| TNM-inf | 48.5 | 24.5 | 28.5 | 93.5 | 45.1 | 88.4 |
| 0-I | 60.2 | 54.9 | 52.2 | 68.1 | 58.0 | 72.0 |
| II | 13.9 | 16.0 | 22.9 | 14.6 | 19.0 | 17.0 |
| III | 7.8 | 11.7 | 6.6 | 6.6 | 12.0 | 4.0 |
| IV | 18.1 | 17.4 | 18.4 | 10.7 | 11.0 | 7.0 |
| **2014-16** | | | | | | |
| No info | 4.2 | 53.2 | 5.6 | 4.2 | 53.2 | 5.6 |
| Partly | 49.0 | 16.1 | 42.9 | 0.5 | 2.3 | 1.6 |
| TNM-inf | 46.8 | 30.7 | 51.5 | 95.3 | 44.5 | 92.7 |
| 0-I | 73.2 | 69.1 | 66.5 | 74.6 | 69.0 | 74.7 |
| II | 11.0 | 12.7 | 17.4 | 14.6 | 14.5 | 15.0 |
| III | 4.1 | 7.9 | 4.3 | 4.0 | 8.2 | 3.4 |
| IV | 11.7 | 10.3 | 11.8 | 6.8 | 8.4 | 7.0 |

Supplementary Table 28. Melanoma skin cancer. Time trends for TNM stages in Denmark, Norway, and Sweden over 2004-2008, 2009-2013 and 2014-2016

| **Melanoma** | Official TNM | | | Using N0M0 for NXMX | | |
|---|---|---|---|---|---|---|
| | Denmark | Norway | Sweden | Denmark | Norway | Sweden |
| **2004-09** | | | | | | |
| No info | 4.8 | 73.7 | 19.9 | 4.8 | 73.7 | 19.9 |
| Partly | 17.4 | 9.4 | 49.0 | 3.0 | 1.7 | 1.1 |
| TNM-inf | 77.9 | 16.9 | 31.1 | 92.2 | 24.5 | 79.0 |
| 0-I | 68.3 | 54.4 | 68.6 | 68.0 | 53.1 | 67.1 |
| II | 16.5 | 31.4 | 25.3 | 18.3 | 35.0 | 28.6 |
| III | 9.8 | 6.1 | 2.8 | 9.1 | 6.2 | 3.0 |
| IV | 5.4 | 8.2 | 3.2 | 4.6 | 5.6 | 1.3 |
| **2009-13** | | | | | | |
| No info | 3.6 | 81.8 | 2.9 | 3.6 | 81.8 | 2.9 |
| Partly | 14.3 | 8.2 | 29.1 | 1.0 | 1.2 | 1.5 |
| TNM-inf | 82.1 | 10.0 | 68.1 | 94.4 | 17.0 | 95.6 |
| 0-I | 74.4 | 70.3 | 73.5 | 72.7 | 68.0 | 71.1 |
| II | 14.1 | 22.2 | 24.0 | 16.6 | 25.3 | 26.5 |
| III | 7.1 | 2.5 | 1.4 | 6.9 | 3.8 | 1.6 |
| IV | 4.4 | 5.0 | 1.1 | 3.8 | 2.9 | 0.8 |
| **2014-16** | | | | | | |
| No info | 5.5 | 5.2 | 2.2 | 5.5 | 5.2 | 2.2 |
| Partly | 15.9 | 75.0 | 16.6 | 2.0 | 0.2 | 0.8 |
| TNM-inf | 78.6 | 19.8 | 81.2 | 92.4 | 94.6 | 97.0 |
| 0-I | 75.3 | 64.8 | 75.1 | 73.5 | 70.6 | 73.3 |
| II | 14.4 | 23.0 | 22.6 | 16.7 | 22.8 | 24.4 |
| III | 7.6 | 6.5 | 1.5 | 7.4 | 5.4 | 1.7 |
| IV | 2.8 | 5.7 | 0.8 | 2.4 | 1.2 | 0.7 |
